# The 3D‐structure, kinetics and dynamics of the *E. coli* nitroreductase NfsA with NADP ^+^ provide glimpses of its catalytic mechanism

**DOI:** 10.1002/1873-3468.14413

**Published:** 2022-07-13

**Authors:** Scott A. White, Andrew J. Christofferson, Alastair I. Grainger, Martin A. Day, David Jarrom, Antonio E. Graziano, Peter F. Searle, Eva I. Hyde

**Affiliations:** ^1^ School of Biosciences University of Birmingham UK; ^2^ School of Science RMIT University Melbourne Australia; ^3^ Institute for Cancer and Genomic Sciences University of Birmingham UK; ^4^ Present address: School of Life and Health Sciences Aston University Birmingham B4 7ET UK; ^5^ Present address: Durham UK; ^6^ Present address: Health Technology Wales Cardiff CF10 4PL UK; ^7^ Present address: Carlsberg Marstons Brewing Company Northampton NN1 1PZ UK

**Keywords:** CB1954, flavoprotein, half‐of‐sites mechanism, NADP(H) binding, nitrofurazone, nitroreductase

## Abstract

Nitroreductases activate nitroaromatic antibiotics and cancer prodrugs to cytotoxic hydroxylamines and reduce quinones to quinols. Using steady‐state and stopped‐flow kinetics, we show that the *Escherichia coli* nitroreductase NfsA is 20–50 fold more active with NADPH than with NADH and that product release may be rate‐limiting. The crystal structure of NfsA with NADP^+^ shows that a mobile loop forms a phosphate‐binding pocket. The nicotinamide ring and nicotinamide ribose are mobile, as confirmed in molecular dynamics (MD) simulations. We present a model of NADPH bound to NfsA. Only one NADP^+^ is seen bound to the NfsA dimers, and MD simulations show that binding of a second NADP(H) cofactor is unfavourable, suggesting that NfsA and other members of this protein superfamily may have a half‐of‐sites mechanism.

## Graphical abstract

*Escherichia coli* NfsA catalyses the reduction of nitroaromatic prodrugs to cytotoxic hydroxylamines by NADPH. We present the crystal structure and kinetics of NfsA with and without NADP^+^ and a model with bound NADPH. Only one NADP^+^ is seen bound to NfsA dimers, while molecular dynamics simulations show that binding of a second NADP(H) is unfavourable, suggesting that NfsA may have a half‐of‐sites mechanism.

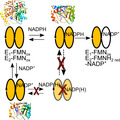

## Abbreviations

**AIC**, Akaike information criterion

**ASU**, asymmetric unit

**MD**, molecular dynamics

**NFZ**, nitrofurazone

*Escherichia coli* NfsA (Nitrofuran sensitive), also known as MdaA (Modulator of drug activity), was discovered due to its activation of nitrofuran antibiotics to give highly reactive, toxic, hydroxylamine derivatives, hence killing bacteria [1]. Mutations in NfsA and the homologous NfsB give bacterial resistance to such antibiotics, but this resistance has not spread *in vivo* as the mutations reduce the fitness of the bacteria [2]. Nitroreductases have also been used to activate nitrofurans in cell ablation studies [3] and proposed for use in cancer gene therapy with the prodrug CB1954 (5 aziridin‐1‐yl‐2,4 dinitrobenzamide) [4], as well as with fluorescent ligands for probes of hypoxia in tumours or of bacterial infection. The role of the NfsA and NfsB enzymes *in vivo* is not known, but they also catalyse the reduction of quinones to quinols and are induced under stress conditions [5, 6, 7] and so are thought to be involved in reducing redox stress. NfsA also reduces the oxidising agent, chromate [8].

**Fig. 1:**
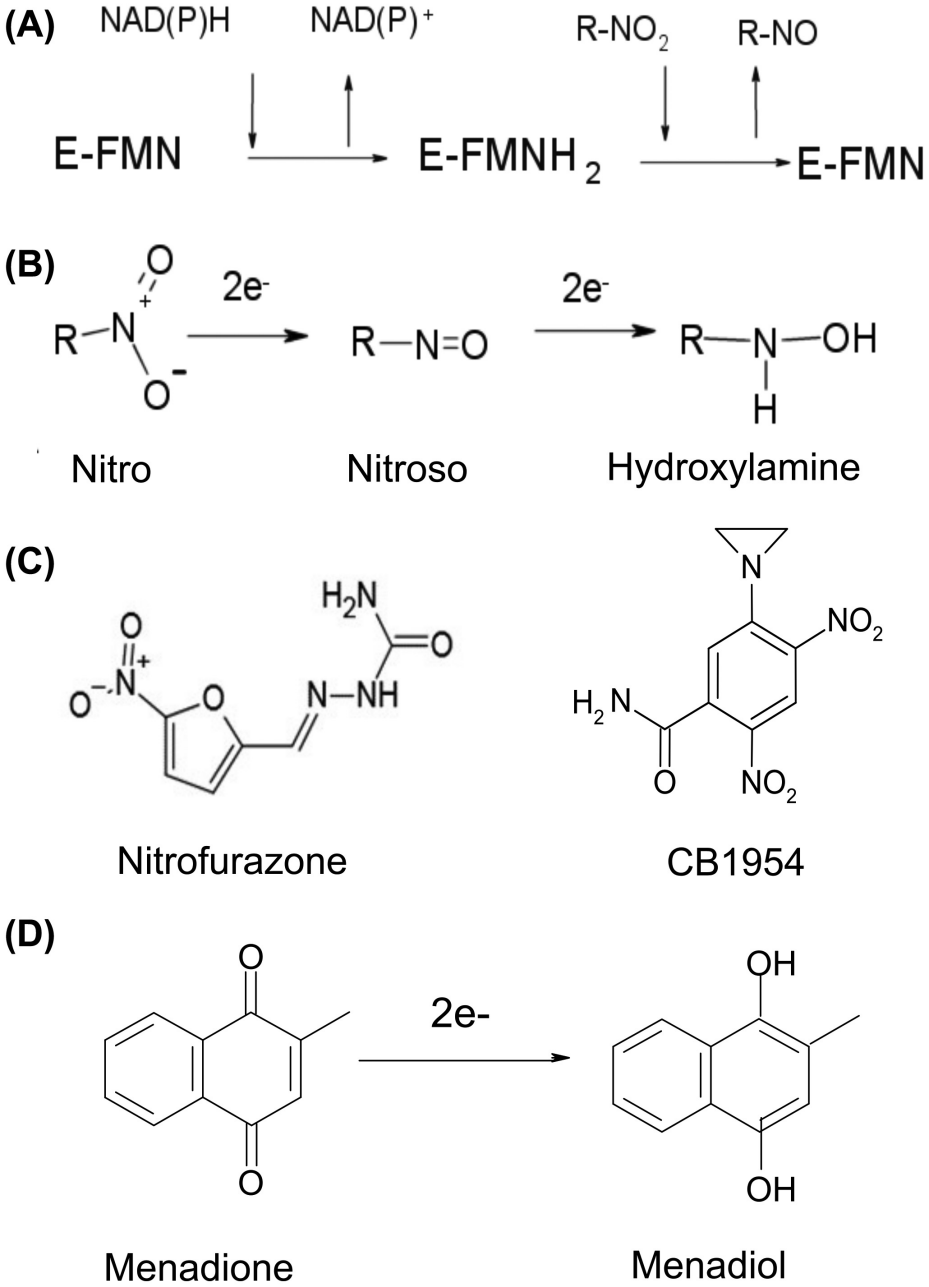
NfsA reaction and selected substrates. (A) Bi‐bi substituted reaction mechanism of nitroreductases. (B) Reduction of nitroaromatic groups to nitroso intermediates and then to hydroxylamines. (C) The nitroaromatic substrates assayed in this paper, left nitrofurazone, right CB1954. (D) The reduction of menadione by NfsA.

NfsA and NfsB are members of a large superfamily of flavoproteins, with more than 24 000 sequences from all domains of life, which catalyse a wide range of reactions, including dehydrogenases, dehalogenases and flavin modification [9]. The proteins have a bi‐bi substituted enzyme mechanism of action whereby the FMN cofactor is initially reduced by NAD(P)H, giving NAD(P)^+^, and then the substrate binds and is reduced [10, 11] (Fig. 1A). NfsA is specific for NADPH, while NfsB can use either NADH or NADPH. The reduction of nitroaromatics gives initial nitroso intermediates, which are more reactive than the nitro compounds and can be reduced to hydroxylamines by NADP(H) in solution as well as by the enzymes [12] (Fig. 1B). Further reduction to the amino group does not occur *in vitro* but does occur *in vivo*, presumably involving a different enzyme.

The structure of NfsA in the absence of ligands was determined by Kobori et al. [13]. The structures of several NfsA homologues have been determined, all in the absence of ligands, apart from FRP a flavin reductase from the bioluminescent *Vibrio harveyi*, which has been crystallised in the presence of the inhibitor, NAD^+^, (2BKJ) [14]. In this structure, the coenzyme is in a folded, ring‐stacked conformation, with the pyrophosphate group close to the FMN, and the N4 of the nicotinamide ring over 10 Å from the FNM N5 and hence in an inactive conformation. We recently determined the structure of NfsA in the presence of the substrates nitrofurantoin and 1,4 benzoquinone, with the product 1,4 hydroquinone and with a second FMN in the active site that acts as an inhibitor [15]. We here present the structure and dynamics of NfsA with NADP^+^ in the active site of the protein and propose a model of the structure with NADPH in the active site in the appropriate orientation for reaction. The crystal structure contains a single cofactor bound to the dimeric protein. This, together with MD simulations and kinetics presented here, leads us to postulate that the protein and its homologues may have a half‐of‐sites mechanism where the binding of NADPH to one active site may be required for the release of NADP^+^ from the other site before reduction of the substrate.

This work complements our previous studies of NfsA complexes [15] and of *E. coli* NfsB with nicotinic acid, a mimic of the cofactor headpiece [16, 17], and also studies of *Enterobacter cloacae* NfsB with NAAD and ligands [18, 19, 20]. Together, the studies may help the design of improved substrates and inhibitors, such as antibiotics and theranostic reagents, for use with this family of proteins.

## Experimental methods

### Protein expression and purification

*Escherichia coli* NfsA was over expressed in *E. coli* BL21 (λDE3) without any tags, from the pET 24 derivative pPS1341A1, encoding NfsA under the control of a T7 promoter, as described in Vass et al. [21]. It was purified as described previously, using ammonium sulphate precipitation, hydrophobic interaction chromatography on Phenyl Sepharose, ion exchange chromatography on Q Sepharose, followed by size exclusion chromatography on Sephacryl 200 or Superdex 75.

Protein concentrations were estimated by Bradford assay [22] or by determining the absorbance at 280 nm where both the protein and the cofactor absorb, and correcting for excess FMN by measuring the absorbance at 454 nm, where only FMN absorbs. The molar absorbances used were 12 200 m ^−1^cm^−1^ for FMN at 454 nm, 20 970 m ^−1^cm^−1^ for FMN at 280 nm and 31 190 m ^−1^cm^−1^ for NfsA at 280 nm, based on its amino acid composition [23].

### Steady‐state enzyme assays

Steady‐state kinetic assays were monitored spectrophotometrically, over 1–2 min, as described previously [24]. Nitrofurazone and CB1954 (Fig. 1C) are poorly soluble in water and were dissolved in 90% DMSO, 10 mm Tris–HCl pH 7.0. Experiments were performed at 25 °C, in 10 mm Tris–HCl pH 7.0, with 4.5% DMSO. Reactions were initiated by the addition of a small quantity of enzyme (~ 10 nm). Reactions were monitored at 420 nm, using molar absorbance change of 4300 m ^−1^cm^−1^ for nitrofurazone and 1200 m ^−1^cm^−1^ for CB1954.

For each reaction, the initial rate (*v*_*i*_) was calculated for a range of concentrations of NADPH or NADH while keeping the concentration of either nitrofurazone or CB1954 constant at 100 μm. The data were fitted to simple Michaelis–Menten curves using non‐linear regression with equal weighting of all points, in sigmaplot14 (Systat Software, San Jose, CA, USA).

For kinetic studies with NADP^+^ the buffer used also included 50 mm NaCl, to minimise the effect of increase in ionic strength on addition of the inhibitor. Initial reaction rates, *v*_*i*_, were measured for a range of concentrations of one substrate [*A*] in the presence of a fixed concentration of the other substrate [*B*], with and without the inhibitor [*I*], at two concentrations of inhibitor. All the data for both substrates were fitted simultaneously to Eqn (1), using non‐linear regression in sigmaplot14. This equation describes inhibition of both halves of the ping‐pong reaction, with *K*_*iA*_ and *K*_*iB*_ being the dissociation constants of the inhibitor in each half reaction. The data were also fitted for inhibition of only one half of the reaction, that is competition with only substrate *A* or only substrate *B* and the fits were compared using the Akaike information criterion (AIC).

(1) $$\frac{v_{i}}{\left[E\right]}=\frac{k_{\mathrm{cat}}\left[A\right]\left[B\right]}{K_{mA}\left[B\right]\left(1+\frac{\left[I\right]}{K_{\mathrm{iA}}}\right)+K_{mB}\left[A\right]\left(1+\frac{\left[I\right]}{K_{\mathrm{iB}}\;}\right)+\left[A\right]\left[B\right]}\;$$

### Stopped flow kinetics

For the reduction of the enzyme, data were collected using a Biologic stopped‐flow apparatus. Solutions containing 10 μm enzyme in one syringe and 25–500 μm NAD(P)H in the other syringe, both in 10 mm Tris–HCl pH 7.0, were mixed rapidly. The absorbance at 340 nm was monitored and fitted to the equation for a first‐order exponential decay, to obtain the pseudo first‐order rate constant *k*_obs_ for enzyme reduction at a given concentration of NAD(P)H. This was then fitted to Eqn (2), below, to obtain the second‐order rate constant for the reduction of the enzyme, *k*/*K*_*d*_, where *k* is the rate of the electron transfer from NADPH to FMN, [*S*] is the concentration of NADPH, and *K*_*d*_ is the dissociation constant of the NADPH‐enzyme complex.

(2) $$k_{\mathrm{obs}}=\frac{k\;\left[S\right]}{K_{d}+\left[S\right]}$$

For the second half reaction, an Applied Photophysics SX20 stopped‐flow spectrophotometer was used, within an anaerobic, Belle technology, glove box. The enzyme was reduced by slow addition of dithionite within the anaerobic chamber and monitored at 454 nm using an Ocean Optics USB2000 + UV–Vis spectrometer, until the solution was almost colourless. It was then mixed either with 50–1000 μm CB1954, in buffer containing 10% DMSO (5% final), or with 25–100 μm menadione (Fig. 1D), in 10 mm Tris–HCl pH 7.0, and the reactions were monitored with a photodiode detector. The data were analysed as for the first half reaction, with [*S*] as the concentration of substrate, monitoring at 454 nm. The dead time of this instrument precluded measuring rates greater than ~ 150 s^−1^.

### X‐ray crystallography

All crystals were grown by a sitting‐drop method. Purified NfsA was concentrated to between 10 and 16 mg·mL^−1^ and then dialysed into 100 mm imidazole, pH 7.0. The mother liquor for NfsA crystallisation contained 100 mm imidazole, pH 7.6, 20% PEG 6000 and 200 mm magnesium formate. To obtain crystals of complexes, 2.5 mm NADP^+^ were added to the mother solution. To cryo‐protect the crystals, they were soaked in mother liquor containing increasing concentrations of DMSO, lowering the concentration of the PEG precipitant alongside each incremental increase in cryo‐protectant. The crystals were then flash‐cooled in liquid nitrogen.

Data were collected at the diamond synchrotron facility, in Oxford, UK. Diffraction images were indexed, integrated and processed using mosflm [25], imosflm [26] or xds [27]. Datasets were combined and scaled using pointless and scala [28] and data quality was assessed using xtriage [29]. Both structures were solved by molecular replacement with phaser [30], using the published NfsA structure pdb entry 1F5V [13] as the starting model. Structures were refined using refmac5 [31] and phenix [29], with the use of ccp4 cloud [32]. Models were built and modified using coot [33]. Final models were validated using molprobity [34, 35] and polygon [36]. The structural figures were drawn using ucsf chimera 1.13.1 [37].

### Modelling and molecular dynamics

#### Structure preparation

Molecular dynamics simulations were based on the NADP^+^ crystal structure in this work, modelled with the protein dimer containing oxidised FMN cofactor in the active site. Input files for NADP^+^ were generated with the Antechamber and Parmchk programs of the amber 12 package [38] using atomic partial charges from gaussian 09 [39] and parameters from the general AMBER force field (GAFF) [40]. Models for FMN were taken from previous work [20]. Final amber prep and frcmod files for NADP^+^ used in this study are provided in the Appendix S1. Hydrogen atoms were added to the amino acids of the protein models using the tleap program of amber 12 according to physiological pH. The amber ff14SB force field [41] was applied to all amino acids. In each case, the system was solvated with a TIP3P water box with a minimum distance from protein surface to box edge of 10 Å and sodium ions to neutralise the overall charge of the system.

#### Molecular dynamics simulations

All simulations were performed using gromacs 2019.3 [42]. acepype [43] was used to convert the topology from amber format to gromacs format. Prior to the MD simulation, a molecular mechanics minimisation was performed on each structure, employing the steepest descent method, with a maximum force convergence criterion of 20 kJ·mol^−1^·nm^−1^. Each simulation was equilibrated by 500 ps of constant pressure MD at 300 K and 1 bar. During minimisation and equilibration, position restraints of 1000 kJ·mol^−1^·nm^−2^ were applied to the protein alpha carbons, aromatic carbons of NADP^+^ and FMN ribityl carbons and phosphorus atoms. Unrestrained simulations, with coordinates saved every 10 ps, were run with temperature maintained at 300 K by a Nose‐Hoover thermostat and pressure maintained at 1 atm with a Parrinello–Rahman barostat. The LINCS algorithm was applied to all bonds to allow a 2‐fs timestep. A 10 Å cut‐off was applied to electrostatic and van der Waals interactions, with the particle‐mesh Ewald scheme applied to long‐range electrostatics. For each system, simulations were performed in an iterative fashion, as described previously [20]. The ptraj tool of amber 12 [38] and vmd 1.9.2 [44] were used for analysis, and ucsf chimera 1.12rc [37] was used for structure modification. All figures were compiled with inkscape.

## Results

### NADP(H) kinetics

**Fig. 2:**
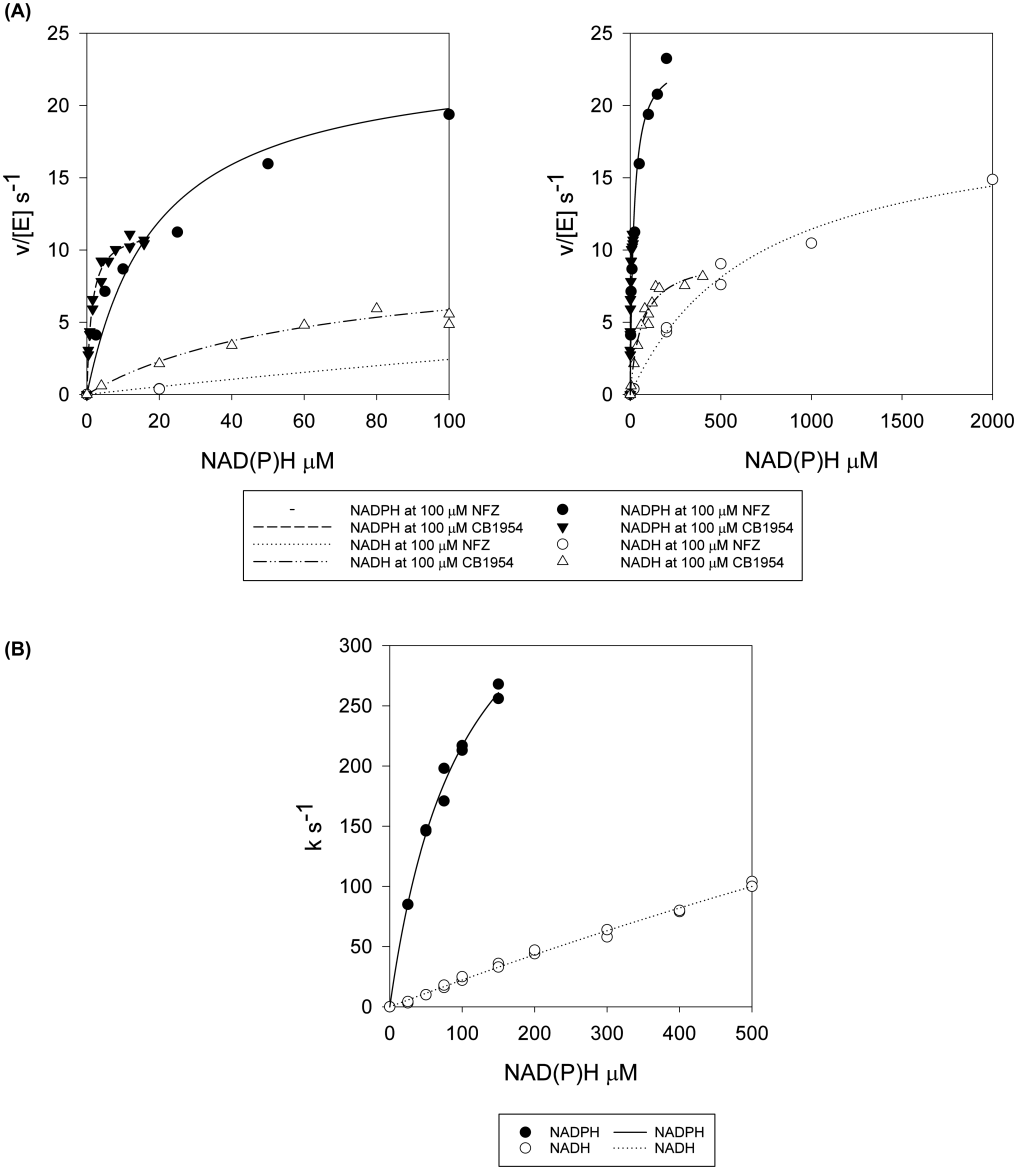
Kinetics of NfsA with NADPH and NADH. (A) Plots of steady‐state rates of NfsA‐catalysed reduction of 100 μm nitrofurazone (circles) or 100 μm CB1954 (triangles) vs NAD(P)H concentration. Black symbols: NADPH used as electron donor; open symbols: NADH used as electron donor. The reactions were performed in a 10 mm Tris pH 7.0 buffer containing 4.5% DMSO, at 25 °C. left‐ inset of data on right. Lines show the curves of the Michaelis parameters in Table 1. (B) Plots of pseudo first‐order rate constants vs NAD(P)H concentration for the reduction of NfsA by NADPH (black circles) or by NADH (open circles) monitored by stopped‐flow. Rate constants were calculated by fitting the absorbance at 340 nm with time after addition of NfsA, to an exponential equation. The reactions were performed in a 10 mm Tris pH 7 buffer at 25 °C with 5 μm NfsA. Lines show the fits to Eqn (2), with the parameters in Table S1.

The major aim of this study was to determine the structure of NfsA in the presence of coenzyme. Previous studies showed that the activity of NfsA at a given concentration of cofactor is much lower with NADH than with NADPH [11, 13, 21]. We therefore reasoned that the 2′ phosphate group of the coenzyme must be important for binding and that NADP^+^ would bind to the protein. To quantify the difference in the activity of the coenzyme, we compared the steady‐state kinetic parameters for NADH or NADPH at a single concentration of nitrofurazone or CB1954 (Fig. 2A). The studies show that the apparent catalytic rate constant, *k*_cat app_, for the reactions with NADH or NADPH is similar but depends on the second substrate. However, with both CB1954 and nitrofurazone, the apparent *K*_*m*_ for NADH is much larger than for NADPH so that the specificity constant *k*_cat_/*K*_*m*_, which controls the rate of reaction at low substrate concentration, is 40–50 fold higher for NADPH than for NADH (Table 1).

**Table 1:** Steady‐state kinetics of NfsA with NADPH or NADH as coenzyme. Steady‐state kinetic data for the reduction of nitrofurazone (NFZ) or CB1954 by *Escherichia coli* NfsA at 10 mm Tris, pH 7.0, 4.5% DMSO, 25 °C, in the presence of NADPH or NADH. Rates were fitted to the Michaelis Menten equation, using non‐linear regression in sigmaplot 14, with equal weighting of points, giving the statistics shown.

Substrate	*k*_cat app_ (s^−1^)	*P*	*K*_m app_ (μm)	*P*	*k*_cat_/*K*_*m*_ (s^−1^·μm ^−1^)	*P*
NADPH (100 μm NFZ)	24 ± 1	< 0.0001	19 ± 4	0.003	1.2 ± 0.2	0.0009
NADH (100 μm NFZ)	20 ± 1	< 0.0001	700 ± 100	0.0004	0.027 ± 0.003	< 0.0001
NADPH (100 μm CB1954)	11.6 ± 0.2	< 0.0001	1. 3 ± 0.1	< 0.0001	8.7 ± 0.6	< 0.0001
NADH (100 μm CB1954)	9.6 ± 0.6	< 0.0001	64 ± 11	0.0002	0.15 ± 0.02	< 0.0001

We also monitored the rate of reduction of the enzyme by each coenzyme using stopped‐flow kinetics (Fig. 2B). For NADPH a nearly full kinetic curve was obtained, giving the rate constant *k* equal to 440 ± 26 s^−1^ and the dissociation constant of the cofactor *K*_*d*_ equal to 100 ± 11 μm; thus, *k*/*K*_*d*_ = 4.3 ± 0.2 μm ^−1^·s^−1^ (Table S1). This maximum rate with NADPH is comparable to the preliminary measurements of Valiauga et al. [45], who obtained rates > 400 s^−1^ at all concentrations of NADPH > 67 μm. For the reduction of the enzyme with NADH, we could not obtain a full kinetic curve as the *K*_*d*_ is very high. However, from the slope of the line, the initial, second‐order rate constant, *k*/*K*_*d*_ for the reaction, is 0.200 ± 0.004 μm ^−1^·s^−1^, that is, 21‐fold lower than that for NADPH. Thus, from both steady‐state and stopped‐flow kinetics, the activity of NfsA with NADPH is an order of magnitude higher than with NADH.

**Fig. 3:**
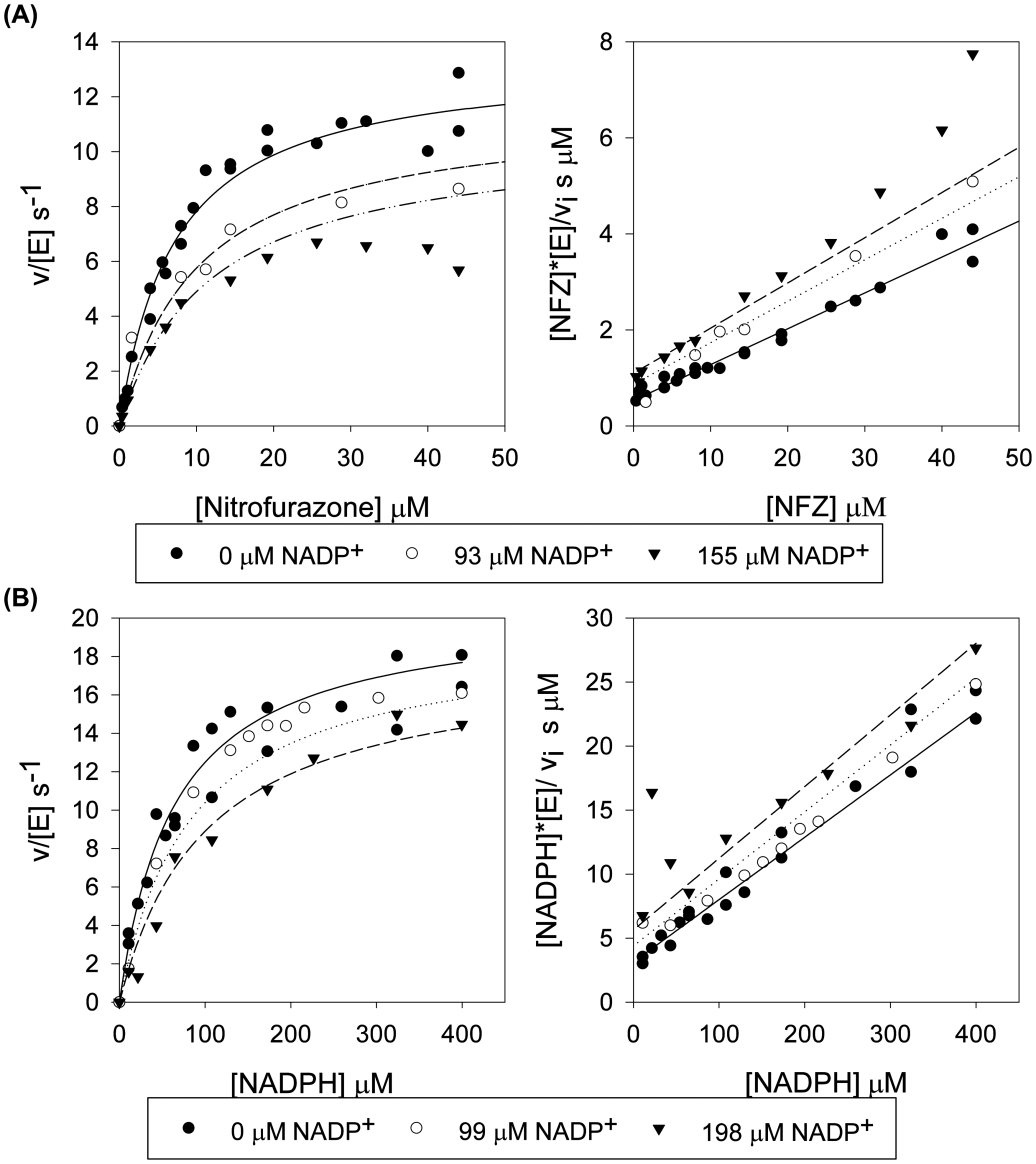
Steady‐state kinetics of NfsA with nitrofurazone and NADPH in the presence or absence of NADP^+^. (A) Reactions measured with 100 μm NADPH, varying the concentration of nitrofurazone. (B) Reactions measured with 99 μm nitrofurazone, varying the concentration of NADPH. All reactions were performed in a 10 mm Tris pH 7.0 buffer containing 50 mm NaCl and 4.5% DMSO, at 25 °C. The symbols show the measured rates and the lines show the simulated Michaelis Menten curves for mixed inhibition (Eqn 1), with *K*_*m* NADPH_ 72 μm, *K*_*m* Nitrofurazone_ 12 μm, *k*_cat_ 23.1 s^−1^, *K*_*i* NADPH_ 249 μm and *K*_*i* Nitrofurazone_ 147 μm as in Table S2. Left: Michaelis Menten plots of initial rate/enzyme concentration *vs* Substrate concentration, Right: Hanes‐Woolf plots of substrate concentration/initial rate *vs* Substrate concentration, normalised by enzyme concentration.

Steady‐state inhibition assays were used to directly measure the binding of NADP^+^ to the enzyme. In these assays, NADP^+^ was found to act as a mixed inhibitor of the reduction of nitrofurazone by the enzyme, with an inhibition constant, *K*_*i*_, of 250 ± 70 μm with respect to NADPH, and a similar *K*_*i*_ with respect to nitrofurazone of 150 ± 50 μm (Fig. 3A,B; Table S2). The inhibition constant of NADP^+^ to the oxidised enzyme is similar to that found by Valiauga et al. [45] using the substrate tertyl, rather than nitrofurazone; however, their analysis, based on each substrate individually, suggested that the *K*_*i*_ to the reduced enzyme is much larger than to the oxidised protein. In our analysis, using all the inhibition data together, while the fit of the data to a simple competition model is good, the Akaike information constant (AIC) for mixed inhibition was 6.3, much lower than the AIC of 16.5, found for simple competition with respect to NADPH. We therefore conclude that NADP^+^ is a mixed inhibitor of the reaction.

### Structure of the free protein

**Fig. 4:**
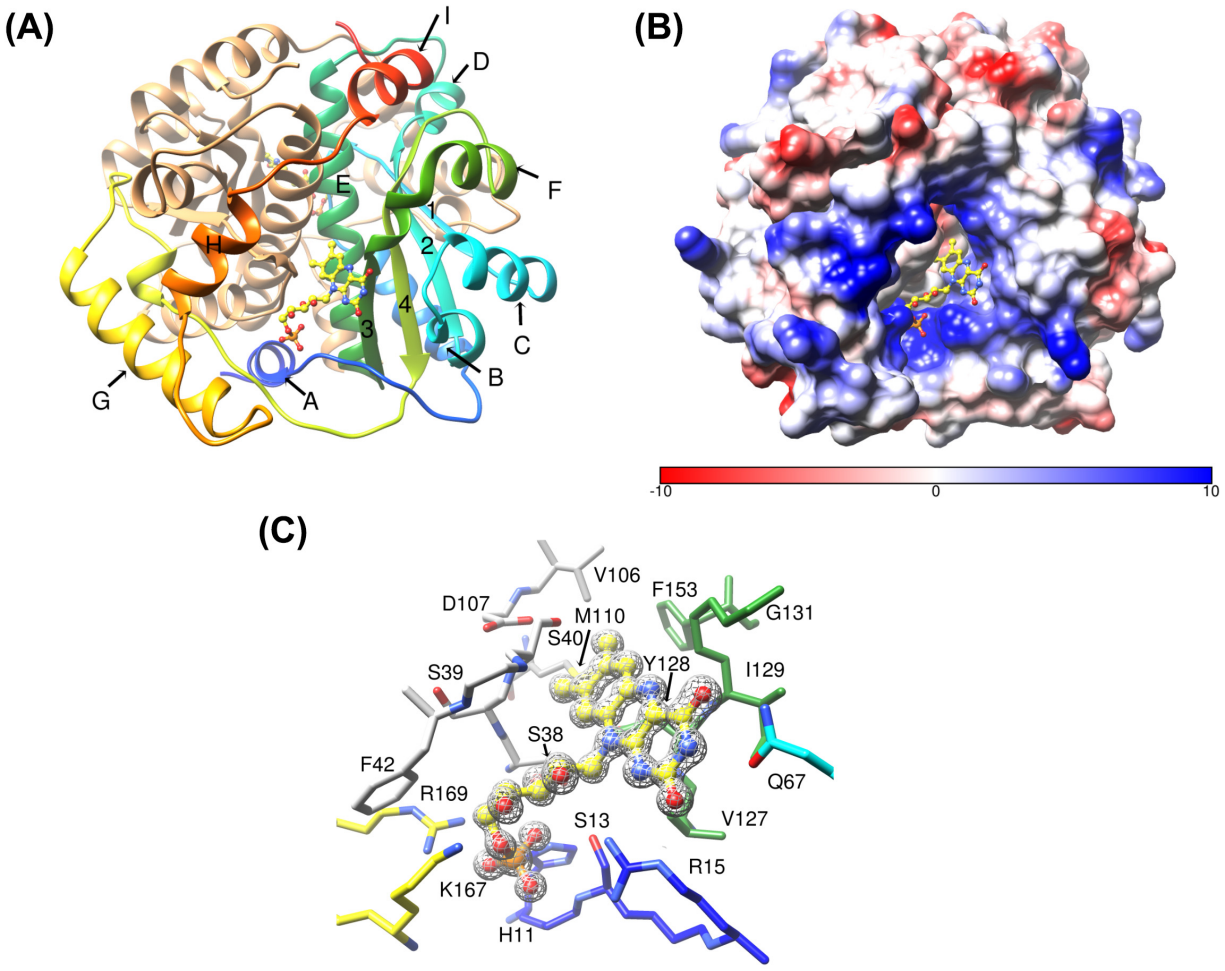
Structure of NfsA in the absence of ligands. (A) Ribbon diagram of NfsA dimer, in the absence of ligands. One subunit is in tan and the other is in rainbow colours blue to red from N‐ to C‐terminus. The helices of this subunit are labelled A–I, and the strands are numbered 1–4. The FMN cofactor is shown as ball and stick, with C atoms in yellow, N blue, oxygen red and phosphorus orange. (B) Surface diagram of NfsA dimer, in the absence of ligands, in the same orientation as (A), coloured from red to blue by coulombic potential. The FMN cofactor is shown as ball and stick, coloured as in (A). (C) The FMN binding site of NfsA. NfsA is shown in the same orientation as in (A). The side chains of residues that interact with the cofactor FMN are shown as sticks, labelled and coloured with the carbon atoms in the same colours as the ribbon (A) and the hetero atoms coloured blue for nitrogen and red for oxygen. The mesh shows the electron density within a radius of 2 Å from the FMN (level 0.44 e) at 2 sigma.

To ensure that any changes we observed in the structure of the protein were due to the presence of NADP^+^, we crystallised the protein under the same conditions in the absence and presence of the cofactor. The structure of NfsA in the absence of ligands was determined with a resolution of 0.96 Å (Table S3) and is shown in Fig. 4.

As with other proteins in this family, the protein is dimeric (Fig. 4A), but there is only one subunit per asymmetric unit (ASU) showing that the dimer has perfect symmetry. Each subunit contains two domains, the core domain of four beta strands surrounded by alpha helices and an excursion domain, residues 165–210 containing two helices, G and H, and long loops. The N‐terminal helix, A, of one subunit interacts with the core domain of the other subunit, as does helix H, and the excursion domain crosses over the dimer interface. The major dimer interface is at helix E, the longest alpha helix in each subunit (residues 100–120).

The two FMN cofactors are ~ 27 Å apart, at either side of the E helices. Each is positioned near the hydrophobic base of a cavity surrounded by positively charged groups (Fig. 4B) and contacts both subunits. The pyrimidine part of isoalloxazine ring forms hydrogen bonds to one subunit, while the dimethylbenzene ring contacts both subunits. There are hydrogen bonds to the FMN phosphate group and to the ribitol chain, as well as extensive van der Waals contacts to both subunits. (Fig. 4C).

This structure is almost identical to that determined by Kobori et al., [13] at 1.7 Å, that is in a different space group, with a dimer in the ASU, but with similar crystal contacts. The structure presented here is at higher resolution, allowing a more detailed comparison of the effects of ligands. The largest difference between the two structures of the free protein is a small change in conformation at the tip of a surface loop between helices G and H, at residues 206–208, which has high B factors in both structures. The RMSD between the Cα positions of the two structures is 0.34 Å across all 240 amino acids.

### NADP ^+^‐bound structure

**Fig. 5:**
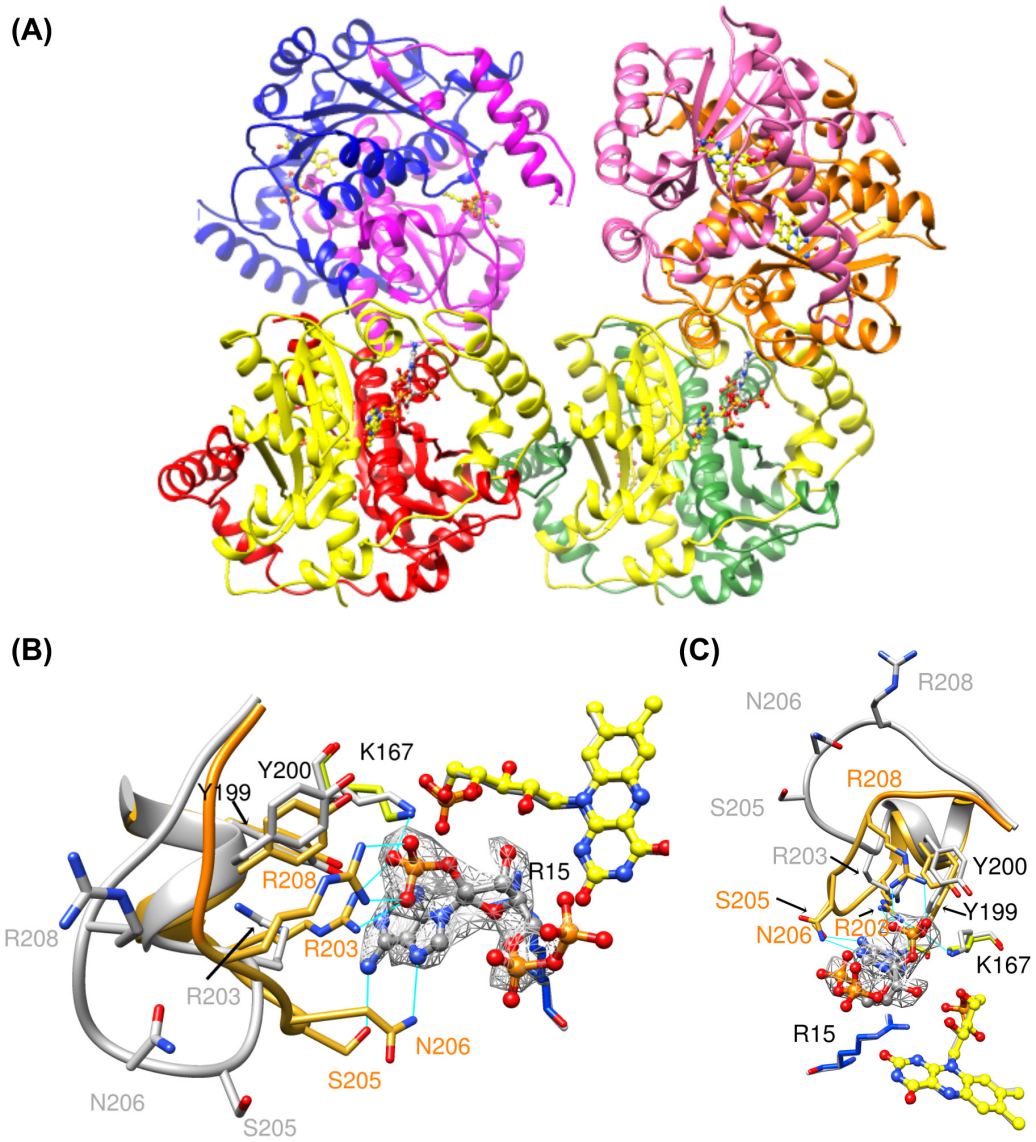
Structure of NfsA with NADP^+^. (A) Ribbon diagram showing the backbone of NfsA and the orientation of the subunits in the structure containing NADP^+^. Subunit A‐red; B and D, containing NADP^+^ in yellow, C‐green, E‐blue, F‐magenta, G‐orange and H‐pink. FMN and NADP^+^ are shown as ball and stick, with the FMN carbon backbone in yellow, the NADP^+^ carbon backbone in grey, nitrogen in blue, oxygen in red and phosphorus in orange. (B and C) Two orientations of the structure of NADP^+^ bound to NfsA, shown overlaid on the structure in the absence of ligands. The NADP^+^ is shown in ball and stick representation with the carbon atoms in grey and the heteroatoms coloured as in (A). Only the adenosine, 2′ phosphate and pyrophosphate moieties are seen. The FMN cofactor of this model is coloured as in (A). The backbone of residues 196–211 and carbon atoms of selected side chains are coloured in yellow/gold for the NADP^+^ bound structure and grey for the structure in the absence of ligand. The heteroatoms of these side chains are coloured blue of nitrogen and red for oxygen. They are labelled in gold for the NADP^+^‐bound structure, grey for the free structure and black for both structures. The mesh shows the electron density within 2 Å of the NADP^+^ ligand at 1 sigma, 0.38 e. Cyan lines show the hydrogen bonds between the protein and the NADP^+^.

In the structure with NADP^+^, determined at 2.1 Å resolution (Table S3), 4 dimers, that is eight subunits, were found in the ASU (Fig. 5A). Only two NADP^+^ moieties were observed, in two different dimers, the other active sites contained only little density, possibly from DMSO. The two subunits with ligand contained very clear electron density for the adenosine half of the cofactor and 2′ phosphate group, but no density for the nicotinamide half of the cofactor nor for one phosphate of the pyrophosphate group (Fig. 5B,C). This suggests that while the adenosine end of the molecule is bound tightly, the nicotinamide end of NADP^+^ is highly mobile. The structure of the protein in each subunit aligns well with each of the other subunits, and with the structure in the absence of ligands, with backbone RMSDs below 0.5 Å, apart from the loop between helices G and H at residues 202–211.

The two subunits with NADP^+^ (subunits B and D) superpose with a backbone RMSD of 0.17 Å. The pyrophosphate moiety has high B factors and does not have any contacts with the protein. The adenosine ribose ring only forms a few van der Waals interactions, mainly to Arg 15. In contrast, the 2′ phosphate group and adenine ring show several interactions with the protein, primarily with the residues in the loop 202–211 (Fig. 5B,C). The 2′ phosphate group has ionic interactions or hydrogen‐bonding interactions with Arg 203, Arg 208 and Lys 167. The adenine ring is shielded in the structure, forming hydrogen bonds to the side chains of Ser 205 and Asn 206 and it is in van der Waals contact with Tyr 199 and Arg 203. Many of the residues in the loop also interact with each other. Tyr 199, Ser 205 and Asn 206 all form hydrogen bonds to Arg 203, while Tyr 200 stacks with Arg 208 and is in van der Waals contact with the 2′ phosphate group of the NADP^+^. The interactions of these residues with the ligand and each other cause the mobile loop between residues 202–211 to move away from its position in the free protein. In the latter, Arg 203, Ser 205, Asn 206 and Arg 208 are out in solution, with Arg 203 forming a salt bridge to Asp165 (Fig. 5B,C).

In the subunits of the structure crystallised in the presence of NADP^+^ that do not contain the ligand, residues 203–209 are either not observed or poorly modelled, presumably due to disorder, apart from in one subunit, subunit H (Fig. S1). In subunit H, this loop is in a different orientation from that in either the free protein or the NADP^+^‐bound subunits. There is also a small change in conformation of 2 residues at the C‐terminal end of Helix H in 2 of the subunits (E and G), resulting in an extra turn. Apart from this, the protein backbone and side chains overlap the structure of the free protein in all the subunits.

### Molecular dynamics simulations of NADP ^+^ binding, model for NADPH binding

**Fig. 6:**
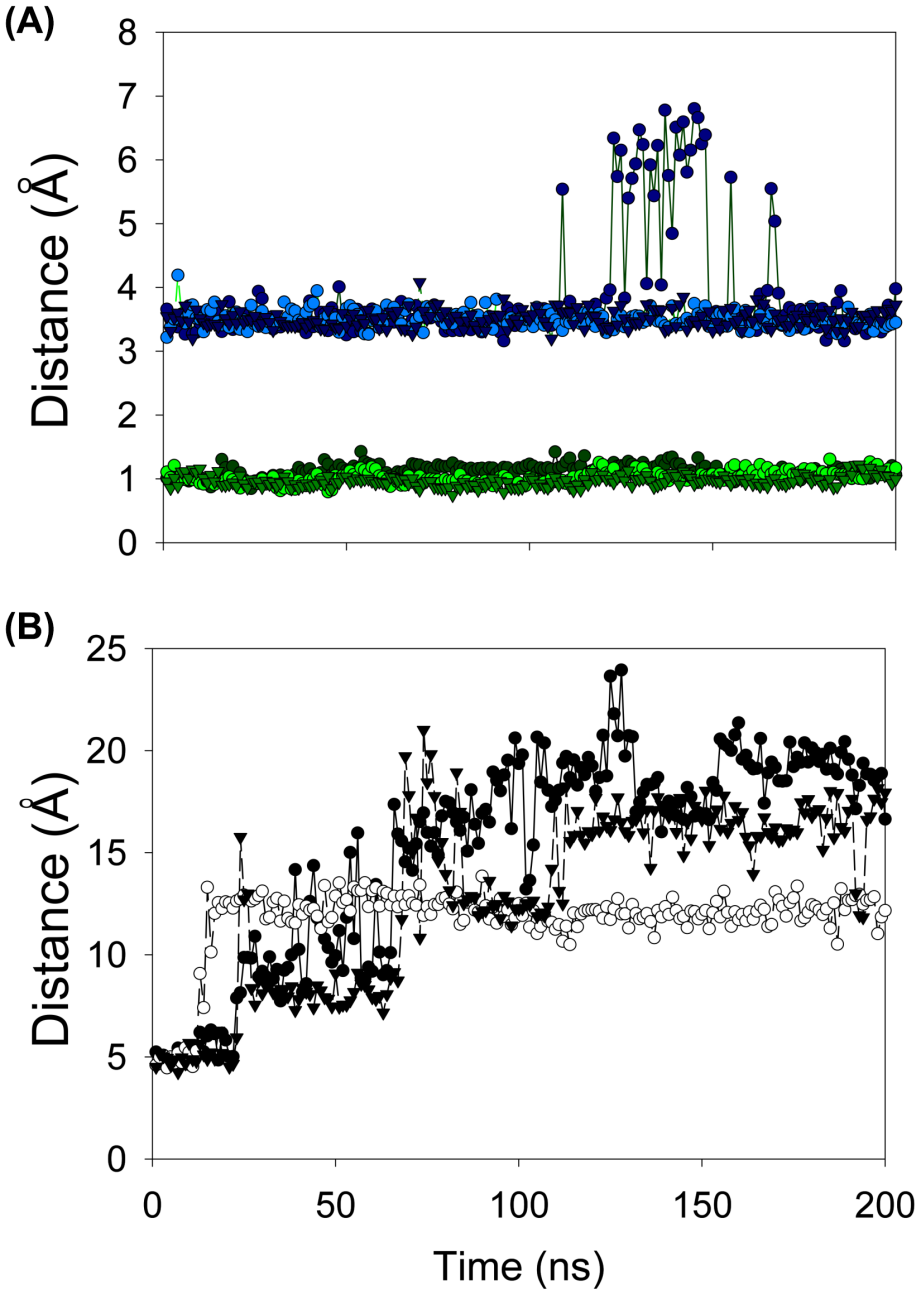
MD calculations of NADP^+^ bound to NfsA. Plot of distances between NADP^+^ and the protein or FMN in oxidised NfsA over 200 ns of MD calculations. Three separate molecular dynamics simulations of NADP^+^ in NfsA were run, based on the crystal structure of the complex, with an intact NADP^+^ in a single active site. (A) The RMSDs of the Cα atoms of the protein backbone from the initial structure are shown in green, the distances between the 2′ phosphate group of NADP^+^ to the K167 side chain amine in blue. The results for each run are shown in different shades and symbols, dark circles for run 1, light circles for run 2 and medium, inverted triangles, for run 3. (B) The distances between the nicotinamide C4 group of NADP^+^ to the FMN N5 position, shown in black circles for run 1, white circles for run 2 and black inverted triangles for run 3.

The structure with NADP^+^ does not show the nicotinamide ring or ribose. However, in NADPH, the hydrogen atom bound at position C4 must come close to the FMN N5 atom for direct hydride transfer in the catalytic mechanism. We have used the structure with NADP^+^ determined above, our structure of nicotinic acid in *E. coli* NfsB [16] and that of NAAD in *E. cloacae* NR [18] to model the complex of *E. coli* NfsA with intact NADP^+^ including the nicotinamide half of the ligand. MD simulations over 200 ns based on the model of NADP^+^ with oxidised NfsA show that the distances between the 2′ phosphate group and the adenine ring to the protein remain largely stable (Fig. 6A, Table S4); in one run, the 2′ phosphate distance to K167 fluctuated at times but returned to the original distance. However, the distance between the C4 of the nicotinamide ring and the FMN N5 is greater than 5 Å and fluctuates greatly (Fig. 6B). This fluctuation confirms that the nicotinamide ring is not seen in the crystal structure due to disorder. However, despite this disorder, the 2′ phosphate group is stable and the binding enthalpy of the cofactor is high with an average value of −68.4 ± 0.8 kcal·mol^−1^ (Table S4).

**Fig. 7:**
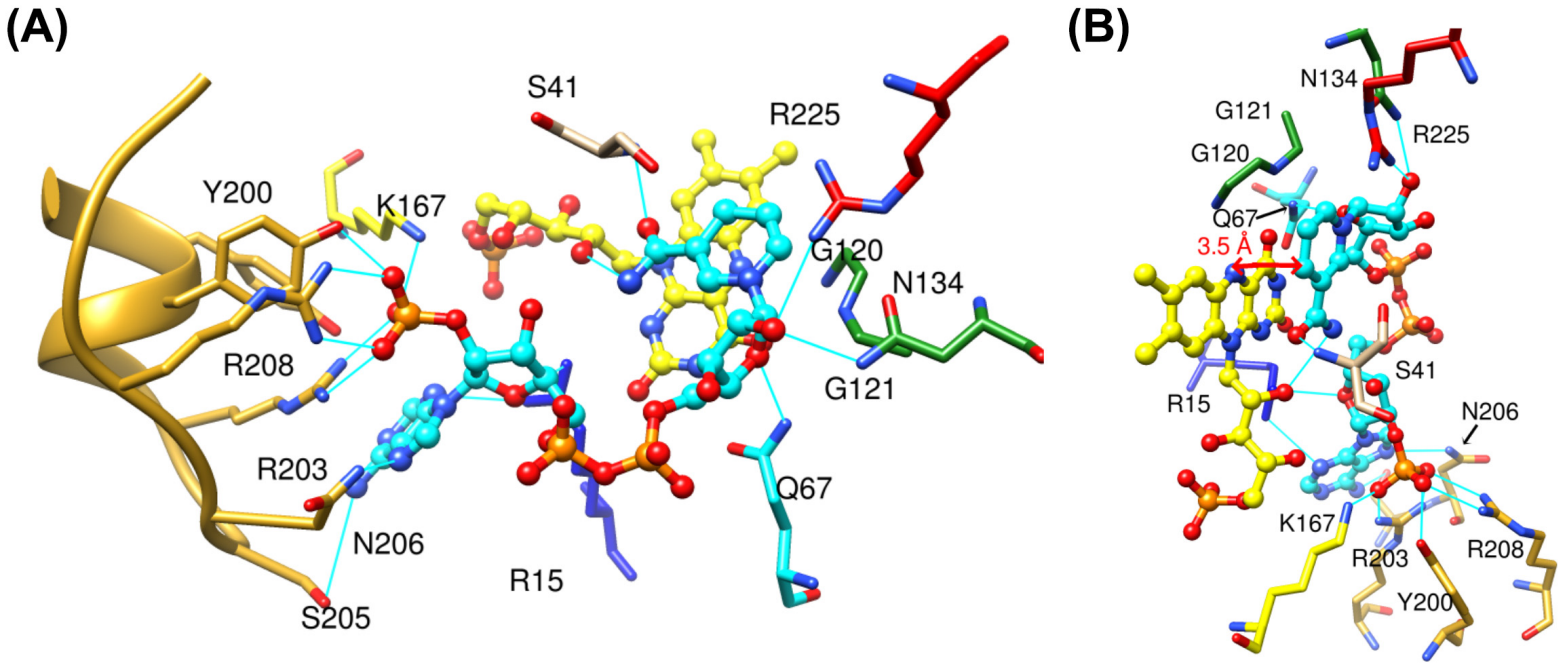
Model of NADPH bound to NfsA. (A,B) Two orientations of NADPH modelled bound to NfsA. The FMN cofactor is shown in ball and stick, coloured as in Fig. 4. NADPH is shown in ball and stick representation with the carbon atoms in cyan and the heteroatoms coloured as for FMN. The side chains that interact with the NADPH are shown as sticks, labelled, with carbons atoms coloured as in the ribbon Fig. 4A, and heteroatoms coloured as in Fig. 4A. Cyan lines show the hydrogen bonding to the ligand. The red arrow shows the distance between the N5 atom of FMN and the C4 of the nicotinamide ring for direct hydride transfer.

We have used the same structures to model NfsA with NADPH (Fig. 7A,B) and to simulate its molecular dynamics. In this model, the nicotinamide N4 was initially placed 3.4 Å from the FMN N5, ready to transfer a hydride group. The nicotinamide ring is stabilised by hydrogen bonds from the amide group to the backbone amide of Ser 41B, (where B denotes the other subunit to the remaining contacts) and to the FMN ribose O2', as well as hydrophobic interactions with Gly 120 and Gly 121. The nicotinamide ribose forms hydrogen bonds to Asn 134, Arg 225 and Gln 67, while the pyrophosphate group and adenine ribose group face into solution and only form hydrogen bonds to solvent.

**Fig. 8:**
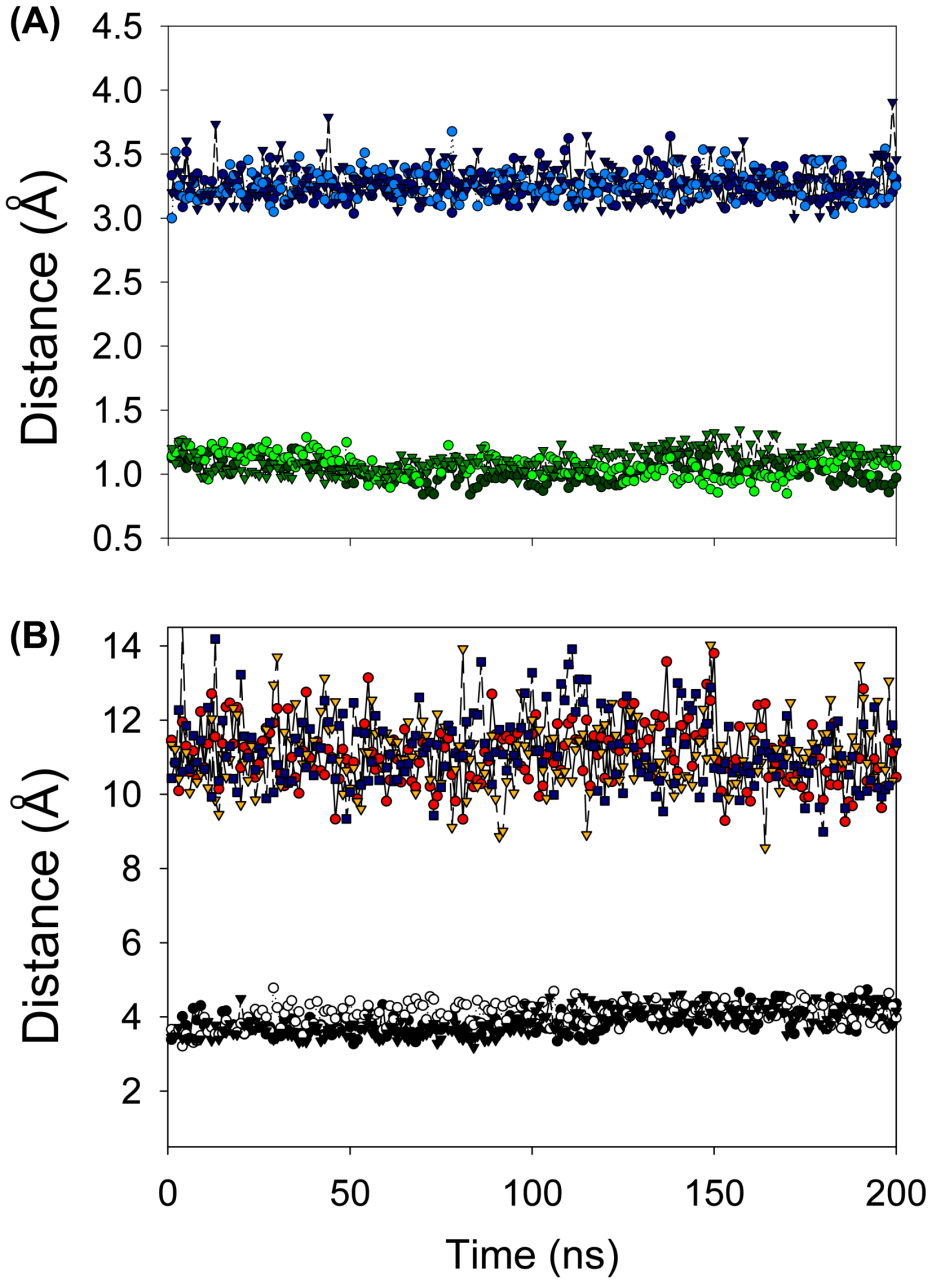
MD calculations of NADPH bound to NfsA. Distances in MD simulations of oxidised NfsA with NADPH modelled bound in the same phosphate pocket as in the crystal structure of NADP^+^ and the head group close to the FMN N5, as shown in Fig. 7A,B. Three separate molecular dynamics simulations were run over 200 ns. (A) The RMSDs of the Cα atoms of the protein backbone from the initial model are shown in green; and the distance between the 2′ phosphate of NADPH to the K167 amine side chain in blue. The results for each run are shown in different shades and symbols, dark circles for run 1, light circles for run 2 and medium, inverted triangles for run 3. (B) The distances between the nicotinamide C4 group of NADPH to the FMN N5 position, shown in black circles for run 1, white circles for run 2 and black inverted triangles for run 3. In colour, the distance between the adenine N6 and S205; red circles for run 1, yellow, inverted triangles for run 2 and blue squares for run 3.

The MD simulations of bound NADPH over 200 ns (Fig. 8A,B, Table S4) show that the distance between the 2′ phosphate and the protein remains stable as before (Fig. 8A) but now, crucially, so does that between the FMN N5 and nicotinamide ring (Fig. 8B), at 3.9 ± 0.3 Å; however, the adenine ring moves away from its initial binding pocket towards Arg 15, which may be a conformational change associated with the formation of the Michaelis complex. The average binding enthalpy observed for NADPH was −85.5 ± 0.6 kcal·mol^−1^.

### Mechanism of reaction

Intriguingly, in the crystal structure of NfsA with NADP^+^, only two of the eight subunits in the ASU have NADP^+^ bound and these are in two different dimers. Attempts to crystallise NfsA in the presence of higher concentrations of NADP^+^ gave poorer quality crystals. MD simulations of symmetrical structures with NADP^+^ in both active sites showed that the coenzyme only remained bound to one of the two sites (Table S5). In all three simulations, all the distances between the coenzyme and the protein were much longer in site 2 than in site 1, the fluctuation of the 2′ phosphate group was larger, and the binding enthalpy was much lower. In addition, the binding enthalpy of the NADP^+^ in site 1 was lower than that seen with only one NADP^+^ bound (maximum −52.6 kcal·mol^−1^, with a second NADP^+^ *cf* average −68.4 kcal·mol^−1^ for a single NADP^+^). Similar effects were seen in simulations with NADPH in both sites (Table S6); the distances to the 2′ phosphate group were longer in site 2 than in site 1 and fluctuated more, the binding enthalpy in site 2 was lower than in site 1, and the binding enthalpy in site 1 was much lower than for a single bound NADPH (maximum −67.4 kcal·mol^−1^ *cf* average –85.5 kcal·mol^−1^); however, the nicotinamide C4 remained close to the FMN N5 in both sites. These calculations show that the binding of a second cofactor to oxidised NfsA is unstable and also destabilises the cofactor bound to the first active site.

**Fig. 9:**
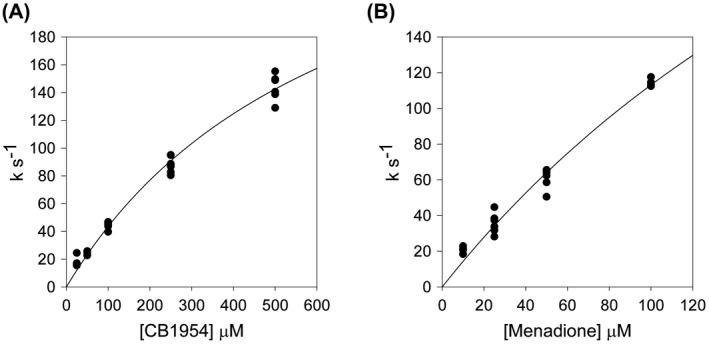
Stopped‐flow kinetics of reduced NfsA with substrates. Plots of pseudo‐first‐order rate constants vs substrate concentration for their reduction by reduced NfsA monitored by stopped‐flow. Rate constants were calculated by fitting the absorbance at 454 nm with time after mixing 5 μm NfsA and substrate, to an exponential equation. Dots show the experimental points, lines show the fit of the data to Eqn (2), with the parameters in Table S1. (A) Reduction of CB1954 in 10 mm Tris pH 7, buffer, 5% DMSO, at 25 °C. (B) Reduction of menadione in 10 mm Tris pH 7, buffer, at 25 °C.

The presence of asymmetric dimers in the crystals and the MD simulations lead us to postulate that the enzyme may have half‐of‐sites reactivity, such that only one subunit can be reduced, or react with NADPH, at one time. In such an alternating half‐site, flip‐flop mechanism, the energy of a cofactor or substrate binding to site 1 can be used to promote dissociation of a product in site 2. This mechanism can therefore be advantageous if product release is rate‐limiting. In previous studies with NfsB, we showed that product release was likely to be rate‐limiting [46]. For NfsA, the maximum global steady‐state rate of reduction of nitrofurazone by NADPH, measured previously, is 30 ± 1 s^−1^, whereas that of CB1954 is 42 ± 1 s^−1^ [15]. These rates are both much slower than rate of reduction of the enzyme by NADPH measured by stopped‐flow above (Fig. 2B), 440 s^−1^. To determine the rate‐limiting step of the reaction, we measured the rate of re‐oxidation of reduced enzyme by CB1954 using stopped‐flow kinetics (Fig. 9A, Table S1). The enzyme was reduced by dithionite and mixed with CB1954 in an anaerobic chamber. The curve of the pseudo first‐order rates *vs* concentration of CB1954 shows a shallow hyperbola. Due to the poor solubility of CB1954 and the dead‐time of the instrument used, we were unable to measure the rate at concentrations higher than 500 μm and so could not get a full rate curve; however, the highest rate measured, 144 ± 9 s^−1^, is already higher than the steady‐state rate, while the fit of the observed data to Eqn (2) gives an extrapolated maximum rate constant 330 ± 30 s^−1^, with a *K*_*d*_ of 660 ± 90 μm. Similar results were obtained with menadione, rather than CB1954, as the oxidant (Fig. 9B). The highest rate we were able to measure was 117 ± 6 s^−1^ at 100 μm menadione. This concentration is well below the estimated *K*_*d*_ (330 ± 115 μm) from the fit of the data to Eqn (2), so giving high error margins for the maximum rate constant 480 ± 140 s^−1^ and *K*_*d*_. However, again, the highest rate observed is much higher than the steady‐state rate of reduction of menadione, measured in our previous study, ~ 12 s^−1^ [15]. From these measurements, both reduction of the enzyme by NADPH and its reoxidation by substrate are much faster than the steady‐state rate, so it is not the chemical step but some other step, most likely product (or oxidised cofactor) release, that is rate‐limiting in NfsA. Hence, an alternating site mechanism using substrate binding to aid product release could be beneficial.

## Discussion

NfsA was known to be much more reactive with NADPH than with NADH. Using steady‐state and stopped‐flow kinetics, we have quantified this to show that NfsA is 20–50 fold more active with NADPH than with NADH. This shows that the 2′ phosphate group of NADPH is important for binding or reaction. The crystal structure of the oxidised enzyme bound to NADP^+^ shows that they interact through the 2′ phosphate group and adenosine part of the molecule; the nicotinamide half of the co‐factor is very mobile, as confirmed by molecular dynamics simulations. This mobility of the charged nicotinamide ring is likely to be because of the high positive charge around the FMN binding site and is not seen in the molecular dynamics simulations of NADPH, where the headgroup is not charged, bound in our model with NfsA.

In the crystal structure, the 2′ phosphate of bound NADP^+^ is in the same position as the phosphate group of FMN bound as an inhibitor in our previous study of NfsA binding to ligands [15] and it is co‐ordinated in a similar way to the same residues. The binding of this phosphate group causes a change in the mobile loop of residues 202–211, to give a phosphate‐binding pocket, containing several charged and hydrogen‐bonding residues, including R203 and R208. As discussed with FMN binding in the previous paper, this mobile loop is not present in NfsB which does not discriminate between NADH and NADPH. R203 is conserved across NfsA homologues, while R or K is often seen at position 208 [47].

In our model of NADPH bound to NfsA (Fig. 7), residues Q67, N134 and R225 interact with the nicotinamide ribose group, while the head group interacts with S41 and the ribitol group of FMN. In our previous crystal study of NfsA, R225 was shown to interact with all of the bound ligands [15], and studies by Ackerley and coworkers have shown that mutation of R225 to a variety of residues reduces *k*_cat_ and affects substrate specificity [48].

The crystal structure of NfsA with NADP^+^ shows asymmetric dimers with only one bound NADP^+^ in each of two dimers (Fig. 5A). The MD simulations show that addition of a second NADP^+^ or NADPH to the dimers weakens the binding of the first cofactor, and the second cofactor does not bind to the mobile loop (Tables S5 and S6). The asymmetrical structure of the NADP^+^‐bound NfsA contrasts with our previous crystal studies of nitrofuratoin, quinone, quinol and a second FMN bound to NfsA which all showed monomers in the ASU, leading to models with symmetrical dimers [15]. Nitrofurantoin, quinone and quinol have full occupancy in the active site. They do not have phosphate groups and do not affect the conformation of the mobile loop of NfsA; however, we note that nitrofurantoin is bound to the oxidised enzyme in an inactive orientation which changes on reduction of the enzyme. Intriguingly, in the FMN‐bound structure, the FMN shows only partial occupancy and the mobile loop of the protein is modelled with 50% in the bound conformation and 50% in the free conformation. For the crystal to show symmetry, the bound and free monomers must be randomly distributed, but it is possible that the structure contains asymmetrical dimers, each with one free and one bound monomer, if each dimer is randomly aligned in the crystal. If only one phosphate‐binding loop in the dimer can be occupied at one time, NfsA is likely to have a half‐of‐sites/alternating‐sites mechanism, where only one active site can be reduced at a time.

Half‐site reactivity has been observed in several redox systems, including members of the PNDOR (pyridine nucleotide disulphide oxidoreductase) family [49, 50, 51], glutamate dehydrogenase [52], glyceraldehyde‐3‐phosphate dehydrogenase [53] and F‐420 H_2_:NADP^+^ oxidoreductase [54]. Apart from the mobile loop, which is far from the active site, there is no difference in the backbone conformation of the protein in the subunits with or without NADP^+^. However, it has been shown that half‐site reactivity need not involve conformational change, just a change in hydrogen‐bonding network, including bound water molecules, at the subunit interface [55]. In NQO1, an FAD‐bound mammalian protein that is unrelated to NfsA but also reduces quinones and CB1954, binding of a ligand in one site of the dimer affects the dynamics of the other site, possibly leading to the observed negative co‐operativity of binding a second ligand [56]. This, or a change in the hydrogen‐bonding network at the active site, is likely to occur in NfsA.

The stopped‐flow studies show that both the rate of reduction of the enzyme by NADPH, in the first half of the reaction and the rate of reduction of the substrate in the second half of the reaction are much faster than the steady‐state rates measured previously [15]. For CB1954, the global steady‐state rate was estimated at 42 s^−1^, based on the rates at a series of CB1954 and NADPH concentrations. For menadione reduction, the *K*_*m*_s of both menadione (11 ± 2 μm) and NADPH (1.8 ± 0.3 μm) are very low, so we previously only reported steady‐state rates at saturating concentrations (100 μm) of each. The same *k*_cat_ and *K*_*m*_ are obtained at 450 μm NADPH as at 100 μm NADPH, confirming the low *K*_*m*_s of both substrates and the limiting *k*_cat_ of 12 ± 1 s^−1^. The much faster rates of reduction and oxidation of the enzyme compared to the steady‐state rates with both substrates show that these steps are not rate‐limiting. While the reduction of nitroaromatic substrates is complicated by a second, fast, reduction step (Fig. 1B), that of quinones is a single 2e^−^ reduction to quinols (Fig. 1D). This suggests that the overall rate of reaction is limited by product release, either of reduced substrate or oxidised cofactor.

The dissociation constant of NADPH from stopped‐flow kinetics, 100 ± 11 μm (Table S1), is similar in magnitude to that of NADP^+^, from either the oxidised enzyme (150 ± 50 μm) or reduced enzyme (250 ± 70 μm), measured by steady‐state inhibition studies (Table S2), suggesting that NADP^+^ does not dissociate readily after reaction. In a half‐of‐sites mechanism, the binding energy of NADPH may allow the conformational change required to release the NADP^+^ from the other active (reduced) site of the dimer. This would then allow the substrate to enter the reduced active site and become reduced in turn.

Our previous kinetic studies suggested that product release is likely to be rate limiting for *E. coli* NfsB [46], while MD simulations gave an active orientation of p‐nitrobenzoate in reduced *Enterobacter cloacae* NR only when the second active site contained an NADH analogue [20]. Half‐site reactivity may be common to the nitroreductase superfamily of proteins. The structural and kinetic studies presented here, together with previous work on substrate binding, should help in the design of improved, mechanism‐based antibiotics and other reagents for NfsA.

## Author contributions

MAD, DJ, AIG and AEG, supervised by EIH and PFS, did kinetics experiments. Crystal trials were done by MAD, DJ, AIG and AEG supervised by SAW. SAW determined the crystal structures. AJC did the modelling and molecular dynamics calculations. The paper was written by EIH, AJC, SAW and PFS.

## Funding

MAD was supported by an MRC PhD studentship; DJ was supported by a BBSRC CASE studentship.

## Supporting information


**Table S1.** Stopped flow kinetics data of oxidised NfsA with NADPH and NADH and of reduced NfsA with CB1954 and menadione.

**Table S2.** Inhibition of nitrofurazone reduction by NADP^+^.

**Table S3.** Crystallographic refinement statistics for free NfsA and NfsA bound to NADP^+^.

**Table S4.** Molecular dynamics simulations of NADP^+^ and NADPH bound to a single active site of oxidised NfsA.

**Table S5.** Molecular dynamics simulations of NADP^+^ bound to both active sites of oxidised NfsA.

**Table S6.** Molecular dynamics simulations of NADPH bound to both active sites of oxidised NfsA.

**Fig. S1.** Overlay of the FMN binding site and mobile loop of each subunit in the NfsA‐NADP^+^ crystal structure.

**Appendix S1.** amber force field prep and frcmod files for NADP^+^ and NADPH used in the molecular dynamics simulations.

## Data Availability

Data Accessibility Research data pertaining to this article is located at figshare.com. The model of NADP^+^ bound to NfsA has been deposited into the ModelArchive as project ma‐iv5o1 (https://modelarchive.org/doi/10.5452/ma‐iv5o1). The model of NADPH bound to NfsA has been deposited into the ModelArchive as project ma‐94ee8 (https://modelarchive.org/doi/10.5452/ma‐94ee8).
