# Supplementary material for: The 3D‐structure, kinetics and dynamics of the E. coli nitroreductase NfsA with NADP + provide glimpses of its catalytic mechanism
Source: FEBS Lett. 2022 Jul 13;596(18):2425–40. doi: 10.1002/1873-3468.14413 (PMC9912195; doi:10.1002/1873-3468.14413)
Supplement: Supplementary file 1 — Table S1. Stopped flow kinetics data of oxidised NfsA with NADPH and NADH and of reduced NfsA with CB1954 and menadione. [file FEB2-596-2425-s002.docx]

| **Substrate** | **k (s^-1^)** | **P** | **K_d_ (µM)** | **P** | **k/K_d_ (s^-1^µM^-1^)** | **P** |
| --- | --- | --- | --- | --- | --- | --- |
| **Oxidised Enzyme** | |  |  |  |  |  |
|  |  |  |  |  |  |  |
| NADPH | 440 ± 26 | <0.001 | 100 ± 11 | <0.001 | 4.3 ± 0.3 | <0.001 |
| NADH |  |  |  |  | 0.200 ± 0.004 | <0.001 |
|  | |  |  |  |  |  |
| **Reduced Enzyme** | |  |  |  |  |  |
|  |  |  |  |  |  |  |
| CB1954 | 330 ± 30 | <0.001 | 660 ± 90 | <0.001 | 0.5 ± 0.02 | <0.001 |
| Menadione | 480 ± 140 | 0.003 | 330 ± 115 | 0.01 | 1.5 ± 0.1 | <0.001 |

Supplementary Table 1: Stopped flow kinetics data of oxidised NfsA with NADPH, NADH and of reduced NfsA with CB1954 and Menadione.

Solutions containing 10 µM enzyme in one syringe and 25- 500 µM NAD(P)H in the other syringe, both in 10 mM Tris-HCl pH 7.0, were mixed rapidly and the absorbance at 340 nm

monitored with time. For the second half reaction, the enzyme was reduced with dithionite and mixed either with 50-1000 µM CB1954, in buffer containing 10% DMSO (giving 5% final) or with 25-100 µM Menadione, and the absorbance at 454 nm monitored. The pseudo first-order rate constants obtained from the exponential fits of the data at each concentration of substrate, were fitted to equation 2.
