# Supplementary material for: The 3D‐structure, kinetics and dynamics of the E. coli nitroreductase NfsA with NADP + provide glimpses of its catalytic mechanism
Source: FEBS Lett. 2022 Jul 13;596(18):2425–40. doi: 10.1002/1873-3468.14413 (PMC9912195; doi:10.1002/1873-3468.14413)
Supplement: Supplementary file 2 — Table S2. Inhibition of nitrofurazone reduction by NADP+. [file FEB2-596-2425-s006.docx]

**Supplementary Table 2**

Inhibition of Nitrofurazone reduction by NADP^+^

| **k_cat_ (s^-1^)** | **P** | **K_m_ (µM)** | **P** | **k_cat_/K_m_**  **(s^-1^µM^-1^)** | **K_i_ (µM)** | **P** |
| --- | --- | --- | --- | --- | --- | --- |
| 23.1 ± 0.9 | <0.001 | 12 ± 1  (K_m_ NFZ)  72 ± 8  (K_m_ NADPH) | <0.0001  <0.0001 | 1.9 ± 0.2  0.32 ± 0.02 | 147 ± 53  (K_i_ NFZ)  249 ± 72  (K_i_ NADPH) | 0.007  0.0009 |

Steady-state kinetic data for NfsA with nitrofurazone and NADPH in the presence of NADP^+^ at 10 mM Tris, pH 7.0, 50 mM NaCl, 4.5% DMSO at 25°C. A series of kinetic experiments were done at different NADP^+^ concentrations, either varying NADPH concentration at 99 µM Nitrofurazone, or varying Nitrofurazone concentration at 100 µM NADPH. The rates of all the reactions were fitted to equation 1, using non-linear regression in Sigmaplot 14, with equal weighting of points, giving the statistics shown
