# Supplementary material for: The 3D‐structure, kinetics and dynamics of the E. coli nitroreductase NfsA with NADP + provide glimpses of its catalytic mechanism
Source: FEBS Lett. 2022 Jul 13;596(18):2425–40. doi: 10.1002/1873-3468.14413 (PMC9912195; doi:10.1002/1873-3468.14413)
Supplement: Supplementary file 3 — Table S3. Crystallographic refinement statistics for free NfsA and NfsA bound to NADP+. [file FEB2-596-2425-s003.docx]

## Supplementary Table 3. X-ray Data collection and refinement statistics.

|  | No Ligand | NADP^+^ |
| --- | --- | --- |
| PDB ID | 7Q0O | 7Z0W |
| Wavelength | 0.928 | 1.00 |
| Resolution range | 41.28- 0.96  (0.985 - 0.96) | 81.45 – 2.06  (2.08-2.06) |
| Space group | C 1 2 1 | P 1 2_1_ 1 |
| Unit cell | 92.904 52.571 65.551  90 134.272 90 | 96.77 110.76 112.25  90 103.84 90 |
| Unique reflections | 133,598 (10998) | 142,123 (9991) |
| Completeness (%) | 96.6 (75.4) | 99.61 (99.89) |
| Mean I/sigma(I) | 16.71 (2.12) | 18.7 (1.95) |
| Wilson B-factor | 11.0 | 31.2 |
| Anisotropy | 0.886 | 0.119 |
| Reflections used in refinement | 132,898 (10923) | 135,159 (4479) |
| Reflections used for R-free | 6,652 (561) | 6,964 (221) (4.9%) |
| R-work | 0.112 (0.2415) | 0.1528 (0.2315) |
| R-free | 0.129 (0.2592) | 0.1866 (0.2656) |
| Number of non-hydrogen atoms | 2,287 | 16,530 |
| macromolecules | 1,959 | 14,777 |
| ligands | 31 | 479 |
| solvent | 298 | 1,275 |
| Protein residues | 240 | 1,899 |
| RMSD bond length (Å) | 0.0125 | 0.013 |
| RMSD bond angles (º) | 1.789 | 1.349 |
| RMSD Chirality | 0.087 | 0.073 |
| RMSD planarity | 0.012 | 0.010 |
| RMSD Dihedral |  | 14.35 |
| F_0_Fc correlation | 0.98 | 0.97 |
| Ramachandranfavoured (%) | 97.1 | 96.42 |
| allowed (%) | 2.9 | 3.52 |
| outliers (%) | 0.00 | 0.05 |
| Rotamer outliers (%) | 1.4 | 1.62 |
| Clashscore | 0 | 2.45 |
| Average B-factor | 21.0 | 42.0 |
| Protein | 19.1 | 34 |
| ligands | FMN 10.2 | ATR 59  FMN 27  Mg 32 |
| solvent | 34.8 | 41.4 |

Statistics for the highest-resolution shell are shown in parentheses.

*R*_work_ = ∑‖*F_o_*| − |*F_c_*|/∑|*F_o_*|, where *F_o_* and *F_c_* are the observed and calculated structure factors for data used for refinement, respectively.

*R*_free_ = ∑‖*F_o_*| − |*F*_c_|/∑|*F_o_*| for 5% of the data not used at any stage of structural refinement.
