# Supplementary material for: The 3D‐structure, kinetics and dynamics of the E. coli nitroreductase NfsA with NADP + provide glimpses of its catalytic mechanism
Source: FEBS Lett. 2022 Jul 13;596(18):2425–40. doi: 10.1002/1873-3468.14413 (PMC9912195; doi:10.1002/1873-3468.14413)
Supplement: Supplementary file 4 — Table S4. Molecular dynamics simulations of NADP+ and NADPH bound to a single active site of oxidised NfsA. [file FEB2-596-2425-s008.docx]

|  | **NADP^+^ crystal structure** | **NADP^+^ MD simulation**  **(single site)** | | | **NADPH MD simulation**  **(single site)** | | |
| --- | --- | --- | --- | --- | --- | --- | --- |
|  |  | **Run 1** | **Run 2** | **Run 3** | **Run 1** | **Run 2** | **Run 3** |
| C4N to FMN N5 (Å) | n.d. | 15 ± 5 | 12 ± 2 | 13 ± 4 | 3.9 ± 0.3 | 4.0 ± 0.3 | 3.9 ± 0.4 |
| P2’ to K167 Nζ (Å) | 3.7 | 3.9 ± 0.9 | 3.5 ± 0.1 | 3.5 ± 0.2 | 3.3 ± 0.1 | 3.3 ± 0.1 | 3.3 ± 0.1 |
| P2’ to R208 Cη (Å) | 4.5 | 4.4 ± 0.2 | 4.3 ± 0.1 | 4.3 ± 0.1 | 4.3 ± 0.1 | 4.3 ± 0.1 | 4.3 ± 0.1 |
| P2’ to R203 Cη (Å) | 4.3 | 4.2 ± 0.1 | 4.3 ± 0.1 | 4.3 ± 0.1 | 4.3 ± 0.1 | 4.3 ± 0.1 | 4.3 ± 0.1 |
| P2’ to Y200 OH (Å) | 4.3 | 4.6 ± 0.9 | 4.6 ± 0.7 | 4.8 ± 0.6 | 4.0 ± 0.1 | 4.0 ± 0.2 | 4.0 ± 0.1 |
| N6 to S205 Oγ (Å) | 3.1 | 4 ± 1 | 3.3 ± 0.6 | 3.4 ± 0.9 | 11.1 ± 0.8 | 11.1 ± 0.8 | 11.1 ± 0.9 |
| N7 to N206 Nδ2 (Å) | 3.2 | 4 ± 1 | 3.2 ± 0.4 | 3.4 ± 0.6 | 9 ± 1 | 8 ± 1 | 9 ± 1 |
| Cα RMSD (Å) |  | 1.2 ± 0.6 | 1.2 ± 0.6 | 1.1 ± 0.6 | 1.3 ± 0.5 | 1.5 ± 0.6 | 1.5 ± 0.6 |
| P2’ RMSF (Å) |  | 1.8 | 1.6 | 1.4 | 1.6 | 1.7 | 1.9 |
| C4N RMSF (Å) |  | 8.7 | 2.8 | 6.0 | 1.5 | 1.4 | 1.6 |
| 202-211 loop Cα RMSD |  | 0.39 ± 0.09 | 0.38 ± 0.09 | 0.35 ± 0.08 | 0.5 ± 0.2 | 0.5 ± 0.1 | 0.5 ± 0.2 |
| 202’-211’ loop Cα RMSD |  | 2.0 ± 0.2 | 1.7 ± 0.2 | 1.7 ± 0.3 | 1.9 ± 0.3 | 2.2 ± 0.3 | 2.2 ± 0.2 |
|  |  |  |  |  |  |  |  |
| Binding Enthalpy (kcal/mol) |  | -66.6 ± 0.8 | -68.8 ± 0.7 | -69.8 ± 0.8 | -83.5 ± 0.6 | -86.0 ± 0.6 | -87.0 ± 0.6 |

**Supplementary Table 4****: Molecular Dynamics simulations of NADP^+^ and NADPH bound to a single active site of oxidised NfsA** Numerical averages and standard deviations for selected distances and energies for molecular dynamics simulations, over 200 ns, of a single NADP^+^ or NADPH bound to an oxidized NfsA dimer. The binding enthalpies are measured over the final 5 ns of the simulation. The 202-211 loop is bound to NADP(H), with 202’-211’ loop in the other subunit of the dimer.
