# Supplementary material for: The 3D‐structure, kinetics and dynamics of the E. coli nitroreductase NfsA with NADP + provide glimpses of its catalytic mechanism
Source: FEBS Lett. 2022 Jul 13;596(18):2425–40. doi: 10.1002/1873-3468.14413 (PMC9912195; doi:10.1002/1873-3468.14413)
Supplement: Supplementary file 5 — Table S5. Molecular dynamics simulations of NADP+ bound to both active sites of oxidised NfsA. [file FEB2-596-2425-s005.docx]

|  | **NADP^+^ crystal structure** | **NADP^+^ MD simulation (both sites)** | | | | | |
| --- | --- | --- | --- | --- | --- | --- | --- |
|  |  | Run 1 | | Run 2 | | Run 3 | |
|  |  | Site 1 | Site 2 | Site 1 | Site 2 | Site 1 | Site 2 |
| C4N to FMN N5 (Å) | n.d. | 10 ± 2 | 15 ± 3 | 18 ± 3 | 16 ± 0.2 | 18 ± 4 | 22 ± 2 |
| P2’ to K167 Nζ (Å) | 3.7 | 4 ± 1 | 11 ± 2 | 5 ± 1 | 12 ± 2 | 6 ± 1 | 17 ± 5 |
| P2’ to R208 Cη (Å) | 4.5 | 4.3 ± 0.1 | 15 ± 3 | 5 ± 2 | 13 ± 4 | 4.4 ± 0.2 | 14 ± 6 |
| P2’ to R203 Cη (Å) | 4.3 | 4.3 ± 0.1 | 22 ± 4 | 4.2 ± 0.2 | 21 ± 3 | 4.0 ± 0.1 | 27 ± 5 |
| P2’ to Y200 OH (Å) | 4.3 | 3.9 ± 0.4 | 13 ± 2 | 5 ± 1 | 13 ± 2 | 5 ± 1 | 16 ± 5 |
| N6 to S205 Oγ (Å) | 3.1 | 4 ± 1 | 13 ± 4 | 4 ± 1 | 18 ± 2 | 5.6 ± 0.9 | 28 ± 4 |
| N7 to N206 Nδ2 (Å) | 3.2 | 4 ± 1 | 18 ± 4 | 4 ± 2 | 20 ± 3 | 5.5 ± 0.9 | 26 ± 4 |
| Cα RMSF (Å) |  | 1.0 ± 0.4 | | 1.1 ± 0.4 | | 1.2 ± 0.5 | |
| P2’ RMSF (Å) |  | 1.77 | 2.57 | 2.16 | 3.54 | 1.89 | 5.28 |
| C4N RMSF (Å) |  | 5.52 | 6.29 | 8.76 | 6.48 | 7.93 | 4.52 |
| 202-211 loop Cα RMSD |  | 0.6 ± 0.1 |  | 0.6 ± 0.3 |  | 0.4 ± 0.1 |  |
| 202’-211’ loop Cα RMSD |  |  | 1.9 ± 0.2 |  | 2.1 ± 0.3 |  | 1.9 ± 0.3 |
| Binding Enthalpy (kcal/mol) |  | -52.6 ± 0.8 | -7.5 ± 0.6 | -36.7 ± 0.6 | -27.8 ± 0.5 | -51.0 ± 0.9 | -23.3 ± 0.5 |

**Supplementary Table 5.** **Molecular Dynamics simulations of NADP^+^ bound to both active sites of oxidised NfsA**

Numerical averages and standard deviations for selected distances and energies for molecular dynamics simulations, over 200 ns, of NADP^+^ bound to both active sites of an oxidized NfsA dimer. The binding enthalpies are measured over the final 5 ns of the simulation.
