# Supplementary material for: The 3D‐structure, kinetics and dynamics of the E. coli nitroreductase NfsA with NADP + provide glimpses of its catalytic mechanism
Source: FEBS Lett. 2022 Jul 13;596(18):2425–40. doi: 10.1002/1873-3468.14413 (PMC9912195; doi:10.1002/1873-3468.14413)
Supplement: Supplementary file 6 — Table S6. Molecular dynamics simulations of NADPH bound to both active sites of oxidised NfsA. [file FEB2-596-2425-s007.docx]

|  | **NADP^+^ crystal structure** | **NADPH MD simulation (both sites)** | | | | | |
| --- | --- | --- | --- | --- | --- | --- | --- |
|  |  | Run 1 | | Run 2 | | Run 3 | |
|  |  | Site 1 | Site 2 | Site 1 | Site 2 | Site 1 | Site 2 |
| C4N to FMN N5 (Å) | n.d. | 5.2 ± 0.9 | 4.2 ± 0.4 | 4.0 ± 0.5 | 4.9 ± 0.9 | 3.7 ± 0.4 | 3.6 ± 0.2 |
| P2’ to K167 Nζ (Å) | 3.7 | 5 ± 1 | 9 ± 3 | 3.9 ± 0.8 | 14 ± 5 | 4.6 ± 0.9 | 5 ± 2 |
| P2’ to R208 Cη (Å) | 4.5 | 4.3 ± 0.1 | 15 ± 3 | 4.2 ± 0.1 | 17 ± 4 | 4.3 ± 0.1 | 13 ± 3 |
| P2’ to R203 Cη (Å) | 4.3 | 4.3 ± 0.1 | 14 ± 6 | 4.3 ± 0.1 | 14 ± 6 | 4.3 ± 0.1 | 14 ± 3 |
| P2’ to Y200 OH (Å) | 4.3 | 4.8 ± 0.9 | 10 ± 3 | 4.8 ± 0.7 | 15 ± 5 | 4.7 ± 0.8 | 5 ± 2 |
| N6 to S205 Oγ (Å) | 3.1 | 3.5 ± 0.8 | 16 ± 4 | 6 ± 2 | 14 ± 4 | 11 ± 1 | 17 ±3 |
| N7 to N206 Nδ2 (Å) | 3.2 | 3.5 ± 0.8 | 19 ± 3 | 6 ± 2 | 16 ± 4 | 11 ± 1 | 20 ± 3 |
| Cα RMSF (Å) |  | 1.1 ± 0.5 | | 1.1 ± 0.5 | | 1.1 ± 0.4 | |
| P2’ RMSF (Å) |  | 1.48 | 3.51 | 1.49 | 6.07 | 1.53 | 2.44 |
| C4N RMSF (Å) |  | 2.21 | 0.95 | 1.69 | 2.32 | 1.51 | 0.78 |
| 202-211 loop Cα RMSD |  | 0.4 ± 0.1 |  | 0.4 ± 0.1 |  | 0.5 ± 2 |  |
| 202’-211’ loop Cα RMSD |  |  | 1.7 ± 0.3 |  | 1.7 ± 0.2 |  | 1.6 ± 0.3 |
| Binding Enthalpy (kcal/mol) |  | -52.4 ± 0.6 | -4 ± 2 | -67.4 ± 0.6 | -30.4 ± 0.5 | -66 ± 1 | -22.0 ± 0.6 |

**Supplementary Table 6.** **Molecular Dynamics simulations of NADPH bound to both active sites of oxidised NfsA** Numerical averages and standard deviations for selected distances and energies for molecular dynamics simulations over 200 ns of NADPH bound to both active sites of an oxidized NfsA dimer. The binding enthalpies are measured over the final 5 ns of the simulation.
