# Supplementary figures and images for: The 3D‐structure, kinetics and dynamics of the E. coli nitroreductase NfsA with NADP + provide glimpses of its catalytic mechanism
Source: FEBS Lett. 2022 Jul 13;596(18):2425–40. doi: 10.1002/1873-3468.14413 (PMC9912195; doi:10.1002/1873-3468.14413)

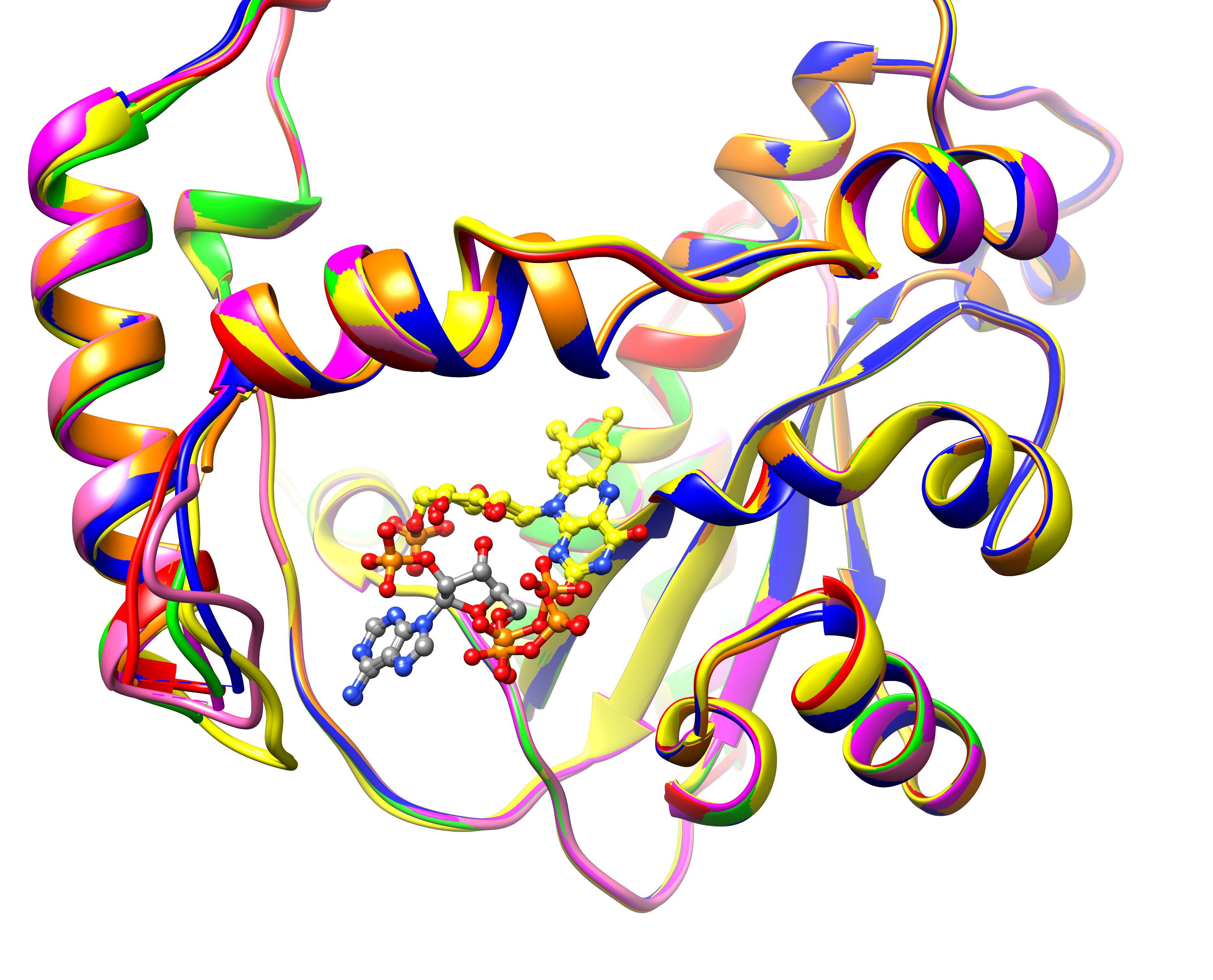

Supplement: Supplementary file 7 — Fig. S1. Overlay of the FMN binding site and mobile loop of each subunit in the NfsA‐NADP+ crystal structure. [file FEB2-596-2425-s004.png]
