# Supplementary material for: The 3D‐structure, kinetics and dynamics of the E. coli nitroreductase NfsA with NADP + provide glimpses of its catalytic mechanism
Source: FEBS Lett. 2022 Jul 13;596(18):2425–40. doi: 10.1002/1873-3468.14413 (PMC9912195; doi:10.1002/1873-3468.14413)
Supplement: Supplementary file 8 — Appendix S1. amber force field prep and frcmod files for NADP+ and NADPH used in the molecular dynamics simulations. [file FEB2-596-2425-s001.doc]

**AMBER force field prep and frcmod files for cofactors**

**NADP^+^ (PAD.prep)**

0 0 2

This is a remark line

molecule.res

PAD INT 0

CORRECT OMIT DU BEG

0.0000

1 DUMM DU M 0 -1 -2 0.000 .0 .0 .00000

2 DUMM DU M 1 0 -1 1.449 .0 .0 .00000

3 DUMM DU M 2 1 0 1.523 111.21 .0 .00000

4 N1 nb M 3 2 1 1.540 111.208 -180.000 -0.727600

5 C6 ca S 4 3 2 1.323 108.368 -13.726 0.680500

6 N6 nh B 5 4 3 1.461 114.372 18.135 -0.871900

7 HN61 hn E 6 5 4 0.860 120.024 62.077 0.395800

8 HN62 hn E 6 5 4 0.860 119.881 -118.002 0.395800

9 C2 ca M 4 3 2 1.358 127.343 -173.033 0.510700

10 H2 h5 E 9 4 3 0.930 120.103 -22.504 0.080900

11 N3 nb M 9 4 3 1.345 119.785 157.372 -0.740300

12 C4 ca M 11 9 4 1.337 119.676 0.005 0.593900

13 C5 ca M 12 11 9 1.351 121.216 -0.228 -0.010800

14 N7 nd M 13 12 11 1.345 108.032 -179.536 -0.548300

15 C8 cc M 14 13 12 1.358 107.531 -0.487 0.185900

16 H8 h5 E 15 14 13 0.930 126.230 -178.321 0.166400

17 N9 na M 15 14 13 1.327 107.631 1.626 -0.111000

18 C1' c3 M 17 15 14 1.484 118.307 -178.922 0.083800

19 O4' os E 18 17 15 1.603 106.867 64.737 -0.457400

20 H1' h2 E 18 17 15 0.972 105.027 -167.990 0.111200

21 C2' c3 M 18 17 15 1.258 120.570 -50.057 0.124100

22 O2' os S 21 18 17 1.471 108.024 -95.176 -0.648500

23 P2' p5 3 22 21 18 1.639 119.622 97.557 1.148100

24 O1P o E 23 22 21 1.523 112.241 10.122 -0.890100

25 O2P o E 23 22 21 1.565 106.823 134.414 -0.890100

26 O3P o E 23 22 21 1.577 105.748 -108.684 -0.890100

27 H2' h1 E 21 18 17 0.970 109.022 31.066 0.026900

28 C3' c3 M 21 18 17 1.668 106.792 153.023 0.150600

29 O3' oh S 28 21 18 1.439 109.357 78.787 -0.683900

30 HO3' ho E 29 28 21 0.978 104.030 40.171 0.433100

31 H3' h1 E 28 21 18 0.969 110.154 -158.770 0.050800

32 C4' c3 M 28 21 18 1.301 106.350 -40.117 0.240300

33 H4' h1 E 32 28 21 0.969 109.665 138.317 0.016200

34 C5' c3 M 32 28 21 1.549 112.878 -101.174 -0.035400

35 H5'1 h1 E 34 32 28 0.970 108.584 -82.158 0.057100

36 H5'2 h1 E 34 32 28 0.970 108.642 158.774 0.057100

37 O5' os M 34 32 28 1.384 112.770 38.289 -0.364200

38 PA p5 M 37 34 32 1.704 121.842 142.786 1.143200

39 O1A o E 38 37 34 1.482 108.624 -153.659 -0.774300

40 O2A o E 38 37 34 1.496 110.695 -34.933 -0.774300

41 O3A os M 38 37 34 1.695 112.096 86.354 -0.468400

42 PB p5 M 41 38 37 1.703 125.879 -10.028 1.143200

43 O2B o E 42 41 38 1.541 109.610 46.252 -0.774300

44 O1B o E 42 41 38 1.517 109.669 166.520 -0.774300

45 O3B os M 42 41 38 1.588 109.335 -73.365 -0.364200

46 C5D c3 M 45 42 41 1.421 116.017 123.015 -0.035400

47 H51 h1 E 46 45 42 1.096 108.819 -0.576 0.057100

48 H52 h1 E 46 45 42 1.098 109.524 -121.357 0.057100

49 C4D c3 M 46 45 42 1.508 107.016 122.656 0.240300

50 C3D c3 3 49 46 45 1.528 115.649 -73.208 0.150600

51 O3D oh S 50 49 46 1.423 110.546 143.825 -0.683900

52 HO3 ho E 51 50 49 0.969 105.593 -97.666 0.433100

53 C2D c3 B 50 49 46 1.531 102.694 -97.074 0.204200

54 O2D oh S 53 50 49 1.421 112.608 -152.968 -0.683900

55 HO2 ho E 54 53 50 0.976 103.603 19.220 0.433100

56 HC2 h1 E 53 50 49 1.093 110.535 83.292 0.026900

57 HC3 h1 E 50 49 46 1.097 110.039 22.094 0.050800

58 HC4 h1 E 49 46 45 1.097 108.958 49.684 0.016200

59 O4D os M 49 46 45 1.446 109.593 166.946 -0.457400

60 C1D c3 M 59 49 46 1.403 109.942 122.469 0.083800

61 HC1 h2 E 60 59 49 1.101 112.263 100.197 0.111200

62 N1N na M 60 59 49 1.513 108.358 -143.364 0.128100

63 C2N ca S 62 60 59 1.343 121.198 14.503 0.038300

64 H12 h4 E 63 62 60 1.081 114.443 0.167 0.181900

65 C6N ca M 62 60 59 1.346 116.853 -163.797 -0.008400

66 H6N h4 E 65 62 60 1.082 115.952 -5.270 0.201800

67 C5N ca M 65 62 60 1.371 119.968 178.004 -0.169900

68 H5N ha E 67 65 62 1.086 119.252 179.212 0.200200

69 C4N ca M 67 65 62 1.384 119.385 -0.236 0.000500

70 H41 ha E 69 67 65 1.083 122.364 -179.936 0.180700

71 C3N ca M 69 67 65 1.390 119.942 0.093 -0.076700

72 C7N c M 71 69 67 1.487 120.838 -179.001 0.697200

73 O8N o E 72 71 69 1.299 114.712 1.000 -0.510200

74 N2 n M 72 71 69 1.224 121.681 -178.507 -0.690900

75 HN21 hn E 74 72 71 1.015 118.055 167.938 0.413700

76 HN22 hn E 74 72 71 1.012 123.414 16.457 0.413700

LOOP

C5 C6

N9 C4

C4' O4'

C1D C2D

C3N C2N

IMPROPER

C5 N1 C6 N6

C6 HN61 N6 HN62

H2 N3 C2 N1

C5 N9 C4 N3

C4 C6 C5 N7

H8 N9 C8 N7

C1' C4 N9 C8

C1D C2N N1N C6N

C3N H12 C2N N1N

C5N H6N C6N N1N

C6N C4N C5N H5N

C5N C3N C4N H41

C7N C2N C3N C4N

C3N N2 C7N O8N

C7N HN21 N2 HN22

DONE

STOP

**frcmod.PAD**

remark goes here

MASS

BOND

ANGLE

DIHE

IMPROPER

ca-nb-ca-nh 1.1 180.0 2.0 Using default value

ca-hn-nh-hn 1.1 180.0 2.0 Using default value

h5-nb-ca-nb 1.1 180.0 2.0 Using default value

ca-na-ca-nb 1.1 180.0 2.0 Using default value

ca-ca-ca-nd 1.1 180.0 2.0 Using default value

h5-na-cc-nd 1.1 180.0 2.0 Using default value

c3-ca-na-cc 1.1 180.0 2.0 Using default value

ca-h4-ca-na 1.1 180.0 2.0 Using default value

ca-ca-ca-ha 1.1 180.0 2.0 General improper torsional angle (2 general atom types)

c -ca-ca-ca 1.1 180.0 2.0 Using default value

ca-n -c -o 10.5 180.0 2.0 General improper torsional angle (2 general atom types)

c -hn-n -hn 1.1 180.0 2.0 General improper torsional angle (2 general atom types)

NONBON

**NADPH (PDH.prep)**

0 0 2

This is a remark line

molecule.res

PDH INT 0

CORRECT OMIT DU BEG

0.0000

1 DUMM DU M 0 -1 -2 0.000 .0 .0 .00000

2 DUMM DU M 1 0 -1 1.449 .0 .0 .00000

3 DUMM DU M 2 1 0 1.523 111.21 .0 .00000

4 N1 nb M 3 2 1 1.540 111.208 -180.000 -0.727600

5 C6 ca S 4 3 2 1.322 108.108 -13.715 0.680500

6 N6 nh B 5 4 3 1.461 114.397 18.037 -0.871900

7 HN61 hn E 6 5 4 0.860 119.970 62.039 0.395800

8 HN62 hn E 6 5 4 0.860 119.934 -117.904 0.395800

9 C2 ca M 4 3 2 1.358 127.579 -173.007 0.510700

10 H2 h5 E 9 4 3 0.929 120.176 -22.578 0.080900

11 N3 nb M 9 4 3 1.346 119.759 157.351 -0.740300

12 C4 ca M 11 9 4 1.337 119.675 -0.073 0.593900

13 C5 ca M 12 11 9 1.350 121.242 -0.149 -0.010800

14 N7 nd M 13 12 11 1.345 108.083 -179.576 -0.548300

15 C8 cc M 14 13 12 1.358 107.513 -0.510 0.185900

16 H8 h5 E 15 14 13 0.929 126.235 -178.320 0.166400

17 N9 na M 15 14 13 1.327 107.616 1.644 -0.111000

18 C1' c3 M 17 15 14 1.483 118.303 -178.927 0.083800

19 O4' os E 18 17 15 1.604 106.856 64.753 -0.457400

20 H1' h2 E 18 17 15 0.971 105.051 -168.058 0.111200

21 C2' c3 M 18 17 15 1.257 120.611 -50.067 0.124100

22 O2' os S 21 18 17 1.472 108.037 -95.173 -0.648500

23 P2' p5 3 22 21 18 1.639 119.630 97.496 1.148100

24 O1P o E 23 22 21 1.524 112.194 10.171 -0.890100

25 O2P o E 23 22 21 1.565 106.809 134.393 -0.890100

26 O3P o E 23 22 21 1.577 105.781 -108.644 -0.890100

27 H2' h1 E 21 18 17 0.971 109.034 31.058 0.026900

28 C3' c3 M 21 18 17 1.669 106.832 152.997 0.150600

29 O3' oh S 28 21 18 1.439 109.352 78.820 -0.683900

30 HO3' ho E 29 28 21 0.981 102.878 37.663 0.433100

31 H3' h1 E 28 21 18 0.969 110.117 -158.724 0.050800

32 C4' c3 M 28 21 18 1.301 106.323 -40.065 0.240300

33 H4' h1 E 32 28 21 0.969 109.664 138.321 0.016200

34 C5' c3 M 32 28 21 1.550 112.822 -101.212 -0.035400

35 H5'1 h1 E 34 32 28 0.970 108.627 -82.139 0.057100

36 H5'2 h1 E 34 32 28 0.970 108.641 158.796 0.057100

37 O5' os M 34 32 28 1.384 112.768 38.322 -0.364200

38 PA p5 M 37 34 32 1.705 121.818 142.768 1.143200

39 O1A o E 38 37 34 1.482 108.577 -153.701 -0.774300

40 O2A o E 38 37 34 1.496 110.728 -34.923 -0.774300

41 O3A os M 38 37 34 1.696 112.053 86.363 -0.468400

42 PB p5 M 41 38 37 1.702 125.939 -10.053 1.143200

43 O2B o E 42 41 38 1.542 109.620 46.251 -0.774300

44 O1B o E 42 41 38 1.517 109.697 166.547 -0.774300

45 O3B os M 42 41 38 1.588 109.311 -73.325 -0.364200

46 C5D c3 M 45 42 41 1.421 116.040 122.971 -0.035400

47 H51 h1 E 46 45 42 1.096 109.080 -0.634 0.057100

48 H52 h1 E 46 45 42 1.096 109.617 -121.307 0.057100

49 C4D c3 M 46 45 42 1.509 107.043 122.636 0.240300

50 C3D c3 3 49 46 45 1.528 115.644 -73.173 0.150600

51 O3D oh S 50 49 46 1.423 110.573 143.765 -0.683900

52 HO3 ho E 51 50 49 0.968 105.191 -94.862 0.433100

53 C2D c3 B 50 49 46 1.530 102.729 -97.083 0.204200

54 O2D oh S 53 50 49 1.421 112.655 -152.946 -0.683900

55 HO2 ho E 54 53 50 0.976 103.400 20.287 0.433100

56 HC2 h1 E 53 50 49 1.092 110.806 83.201 0.026900

57 HC3 h1 E 50 49 46 1.097 110.127 22.420 0.050800

58 HC4 h1 E 49 46 45 1.098 108.799 49.373 0.016200

59 O4D os M 49 46 45 1.445 109.650 166.990 -0.457400

60 C1D c3 M 59 49 46 1.403 109.991 122.451 0.083800

61 HC1 h2 E 60 59 49 1.102 111.839 99.159 0.111200

62 N1N nh M 60 59 49 1.512 108.399 -143.332 0.156800

63 C2N c2 S 62 60 59 1.344 121.185 14.458 -0.079900

64 H12 h4 E 63 62 60 1.080 114.781 -0.178 0.159100

65 C6N c2 M 62 60 59 1.345 116.860 -163.761 -0.258400

66 H6N h4 E 65 62 60 1.085 115.919 -4.218 0.195100

67 C5N c2 M 65 62 60 1.372 119.962 178.007 -0.171300

68 H5N ha E 67 65 62 1.088 119.448 178.765 0.117800

69 C4N c3 M 67 65 62 1.383 119.359 -0.316 0.043500

70 H41 hc E 69 67 65 1.124 110.059 -126.581 0.025500

71 H42 hc E 69 67 65 1.122 110.083 127.224 0.025500

72 C3N ce M 69 67 65 1.389 119.966 0.171 -0.135100

73 C7N c M 72 69 67 1.487 120.841 -179.000 0.626500

74 O8N o E 73 72 69 1.300 114.697 0.931 -0.586100

75 N2 n M 73 72 69 1.223 121.714 -178.497 -0.901600

76 HN21 hn E 75 73 72 1.015 117.467 164.585 0.391300

77 HN22 hn E 75 73 72 1.012 121.509 20.439 0.391300

LOOP

C5 C6

N9 C4

C4' O4'

C1D C2D

C3N C2N

IMPROPER

C5 N1 C6 N6

C6 HN61 N6 HN62

H2 N3 C2 N1

C5 N9 C4 N3

C4 C6 C5 N7

H8 N9 C8 N7

C1' C4 N9 C8

C3N H12 C2N N1N

C5N H6N C6N N1N

C6N C4N C5N H5N

C2N C4N C3N C7N

C3N N2 C7N O8N

C7N HN21 N2 HN22

DONE

STOP

**frcmod.PDH**

remark goes here

MASS

BOND

ANGLE

DIHE

c2-ce-c3-c2 1 0.000 0.000 2.000 same as X -c2-c3-X

c2-ce-c3-hc 1 0.380 180.000 -3.000 same as hc-c3-c2-c2

c2-ce-c3-hc 1 1.150 0.000 1.000 same as hc-c3-c2-c2

c2-c3-ce-c 1 0.000 0.000 2.000 same as X -c2-c3-X

hc-c3-ce-c 1 0.000 0.000 2.000 same as X -c2-c3-X

IMPROPER

ca-nb-ca-nh 1.1 180.0 2.0 Using default value

ca-hn-nh-hn 1.1 180.0 2.0 Using default value

h5-nb-ca-nb 1.1 180.0 2.0 Using default value

ca-na-ca-nb 1.1 180.0 2.0 Using default value

ca-ca-ca-nd 1.1 180.0 2.0 Using default value

h5-na-cc-nd 1.1 180.0 2.0 Using default value

c3-ca-na-cc 1.1 180.0 2.0 Using default value

ce-h4-c2-nh 1.1 180.0 2.0 Using default value

c2-h4-c2-nh 1.1 180.0 2.0 Using default value

c2-c3-c2-ha 1.1 180.0 2.0 Using default value

c -c2-ce-c3 1.1 180.0 2.0 Using default value

ce-n -c -o 10.5 180.0 2.0 General improper torsional angle (2 general atom types)

c -hn-n -hn 1.1 180.0 2.0 General improper torsional angle (2 general atom types)

NONBON
